# Systems-Level Decoding of Redox Homeostasis in Cancer: From ROS Biology to Therapeutic Vulnerabilities

**DOI:** 10.3390/molecules31203789

**Published:** 2026-10-21

**Authors:** Yutian Zhang, Qikang Wang, Lijiao Zhao, Rugang Zhong, Guohui Sun, Ning Lin

**Affiliations:** 1 Beijing Key Laboratory of Environmental and Viral Oncology, College of Chemistry and Life Science, Beijing University of Technology, Beijing 100124, China; zhangyutian@emails.bjut.edu.cn (Y.Z.); wangqikang@emails.bjut.edu.cn (Q.W.); zhaolijiao@bjut.edu.cn (L.Z.); lifesci@bjut.edu.cn (R.Z.); 2 The Department of Clinical Laboratory, The Affiliated Huai’an No. 1 People’s Hospital of Nanjing Medical University, Huai’an 223300, China

**Keywords:** ROS, oxidative stress, tumor metabolism, tumor microenvironment, redox-targeted therapy

## Abstract

Reactive oxygen species (ROS) generated during cellular respiration are highly reactive toward proteins, lipids, and DNA. In the context of malignancy, excessive ROS can cause genomic damage, induce mutations, reprogram metabolic pathways, and sustain pro-survival signaling, thereby collectively promoting tumor progression and adaptive fitness. To cope with elevated oxidative stress, tumor cells upregulate antioxidant systems, including the Nrf2–Keap1 axis, the GSH/GPX4 system, and the Trx/TrxR pathway, which collectively maintain ROS within a tolerable range. This redox balance supports malignant growth while simultaneously creating vulnerabilities that may be therapeutically exploited. Current therapeutic strategies can be broadly categorized into two approaches: elevating ROS to levels sufficient to trigger regulated cell death and disrupting redox homeostasis to enhance tumor sensitivity to conventional therapies. However, the spatiotemporal heterogeneity of ROS generation and distribution presents a major challenge to precise therapeutic intervention. Therefore, identifying critical regulatory nodes and establishing robust, quantifiable biomarkers are essential for improving therapeutic precision. Here, we synthesize current knowledge of ROS biology and redox regulation across diverse tumor types, with particular emphasis on therapeutic combinations that integrate redox modulation with metabolic targeting, DNA damage response, and immune activation. Moving forward, quantitative monitoring, systems-level integration, and personalized treatment strategies will be critical for translating redox-based approaches from mechanistic investigation into clinical practice.

## 1. Introduction

In 2024, global cancer diagnoses reached nearly 20 million, while cancer-related deaths surpassed 9.7 million, according to the latest statistics released by the World Health Organization (WHO) [1]. Population aging and changing lifestyles are key drivers of this growing burden, making cancer an increasingly pressing public health challenge. Against this backdrop, therapeutic strategies that exploit ROS in tumor cells have attracted growing interest. Historically, oxidative stress was regarded as merely a byproduct, or at best a coincidental feature, of tumorigenesis. However, a growing body of evidence now positions ROS not as metabolic waste, but as central regulators of tumor signaling, metabolic reprogramming, and stromal remodeling [2]. Through the reversible oxidation of cysteine residues in proteins, ROS modulate the activity of kinases, phosphatases, and transcription factors, thereby influencing cell-fate decisions at the molecular level [3]. Such redox signaling has therefore become integral to understanding tumor plasticity. Moreover, elevated ROS levels are characteristic of both solid tumors and hematological malignancies [4]. Multiple sources contribute to this oxidative state, including electron leakage from the mitochondrial respiratory chain, NADPH oxidase (NOX) activation, increased oxidative folding burden in the endoplasmic reticulum (ER), peroxisomal metabolic reprogramming, and inflammation-driven microenvironments [5,6]. Rather than eliminating ROS outright, tumor cells establish a multilayered antioxidant network encompassing the Nrf2–Keap1 axis, GSH/GPX4 and Trx/TrxR systems, and NADPH-generating pathways, thereby maintaining ROS within a pro-signaling yet non-cytotoxic range [6]. The redundancy embedded within these systems provides redox flexibility, allowing tumors to exploit the functional benefits of ROS while preventing lethal oxidative damage. This tight regulation suggests that tumor dependence on ROS reflects an adaptive homeostatic state rather than merely the consequence of oxidative injury. The functional effects of ROS, however, remain highly context-dependent [7]. Sustained low-to-moderate ROS signaling promotes genomic instability, activates survival pathways such as MAPK, PI3K/AKT, and NF-κB, and drives malignant progression through the remodeling of tumor metabolism and the immune microenvironment [8]. At higher concentrations, ROS trigger apoptosis, necrosis, ferroptosis, and immunogenic cell death [9]. Whether ROS function as survival signals or death triggers depends largely on two factors: their subcellular site of production and the intensity of oxidative stress. Tumor cells therefore do not simply eliminate ROS; rather, they fine-tune both their concentration and spatial distribution within a permissive range.

Despite decades of investigation, ROS-directed therapies, encompassing both antioxidant and pro-oxidant strategies, have yielded notoriously inconsistent clinical outcomes. In certain malignancies, antioxidant treatment has even been associated with accelerated disease progression rather than tumor suppression [10]. These seemingly contradictory findings highlight a fundamental gap in our understanding: ROS are not a uniform entity, but rather a multidimensional signaling system shaped by their source, subcellular localization, and dose-dependent effects.

The present review examines the dual roles of ROS in tumorigenesis, malignant progression, and treatment sensitivity, tracing their dynamics from production to clearance. Particular emphasis is placed on how oxidative stress pathways are integrated at the regulatory level and on reassessing the theoretical rationale and translational challenges of existing ROS-modulating strategies. By framing ROS within a dynamic homeostatic context, this review aims to provide a conceptual foundation for next-generation interventions with greater selectivity and spatiotemporal precision.

## 2. A Brief Overview of ROS Generation and Resistance in Cancer

### 2.1. Production of Endogenous and Exogenous ROS in Tumor

**Figure 1:**
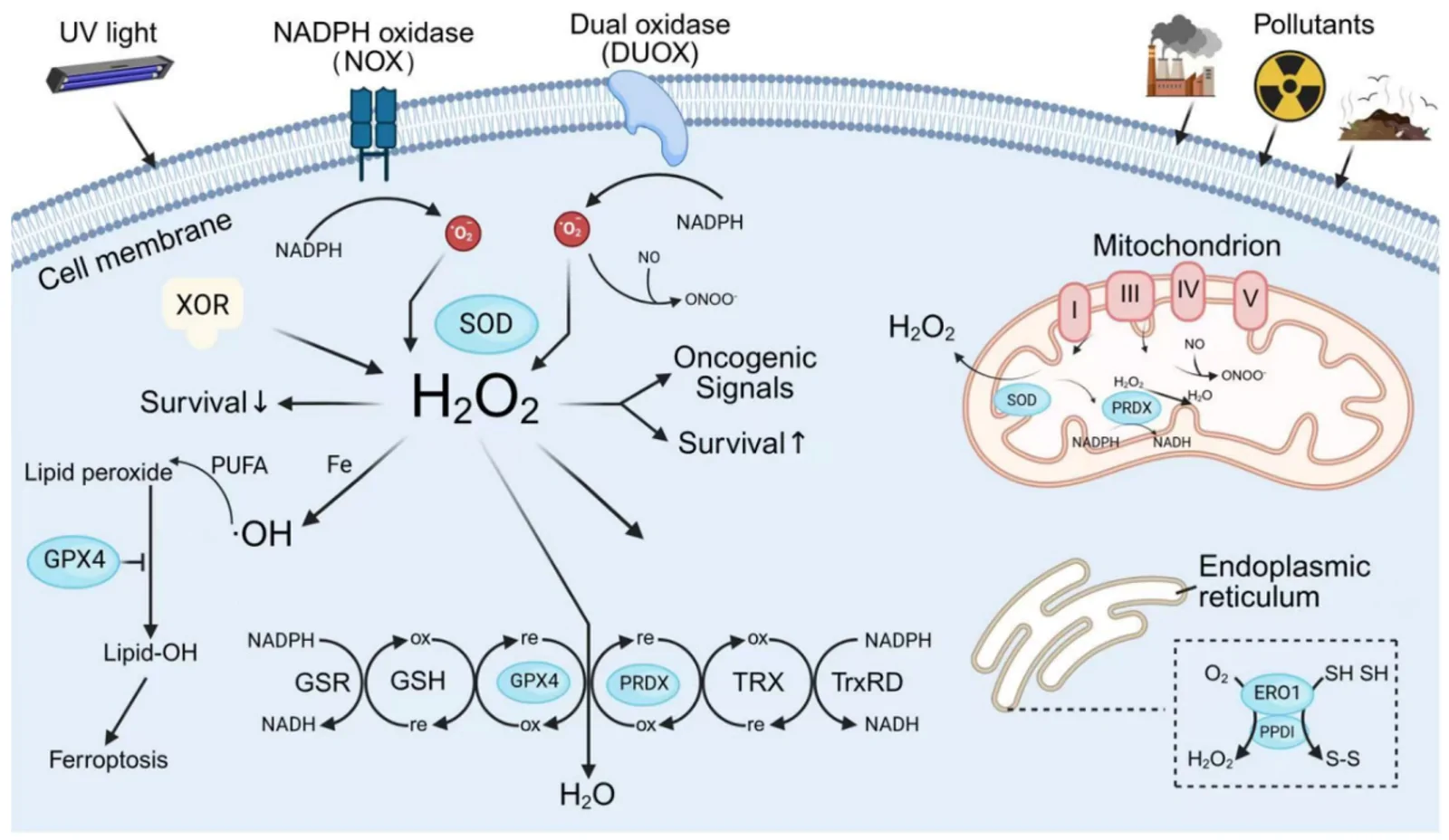
The sources of cellular ROS are remarkably diverse, ranging from endogenous metabolic processes to exogenous environmental insults. Endogenous sources are constituted by the respiratory chain complexes of mitochondria, along with NOX/DUOX, XOR, and ER-mediated oxidative protein folding, while exogenous ones derive from UV light and environmental contaminants. SODs catalyze the conversion of superoxide anion (O_2_·^−^) to H_2_O_2_. Depending on the cellular context, this H_2_O_2_ may engage in redox signaling that promotes tumor cell survival, or it may undergo Fenton chemistry to generate hydroxyl radicals (·OH), reactive species capable of inflicting oxidative damage on cancer cells. Excessive lipid peroxidation can drive ferroptosis. Antioxidant systems, including GSH-GPX4, PRDX, and TRX/TrxR pathways, normally scavenge ROS to limit such damage and preserve redox balance. The balance between ROS production and antioxidant capacity thus plays a critical role in dictating cellular outcomes and tumor behavior under various conditions.

In malignancy, ROS exert opposing effects: at moderate levels, they support survival signaling and adaptive responses, whereas once their concentration exceeds a critical threshold, they become cytotoxic [11]. Most tumor cells exist in a persistently high-ROS state, with endogenous ROS production arising from four principal compartments: mitochondria, the plasma membrane-associated NADPH oxidase (NOX/DUOX) system, the oxidative folding network of the endoplasmic reticulum (ER), and peroxisomal oxidases [5]. These redox-generating sources are summarized in Figure 1. They do more than sustain intrinsic signaling within cancer cells; they actively participate in reshaping the surrounding TME.

#### 2.1.1. Mitochondria

Mitochondria constitute one of the principal endogenous sources of ROS within cells. When electrons leak from the electron transport chain during oxidative phosphorylation, molecular oxygen is partially reduced to superoxide (O_2_·^−^). Where this O_2_·^−^ is generated influences its subsequent fate. In the mitochondrial matrix, for instance, the manganese-dependent enzyme SOD2 converts O_2_·^−^ to H_2_O_2_ [12]. H_2_O_2_ may then be further reduced to water via the PRDX and glutathione peroxidase (GPX) systems. This reduction step depends on thioredoxin (Trx) and reduced glutathione (GSH) as electron donors, and its efficiency helps to limit oxidative damage to proteins and lipids. Under certain conditions, a fraction of O_2_·^−^ may instead react with intramitochondrially generated nitric oxide (NO) to form peroxynitrite (ONOO^−^) [13], which can contribute to nitrosative stress and mitochondrial dysfunction (see Figure 1).

A growing body of literature reports that disruption of mitochondrial membrane structure and morphological abnormalities lead to the excessive production of ROS. For example, Shao and co-workers found that in hepatocellular carcinoma (HCC), the Src kinase phosphorylates the tyrosine residues at positions Y34 and Y77 of the inhibitor PHB2, disrupting the binding of PHB2 to cardiolipin and mediating the dissociation of the PHB1/2 complex, thereby triggering mitochondrial cristae remodelling and ultimately promoting the abnormal accumulation of mitochondrial ROS. Functional validation indicates that a PHB2 phosphorylation-resistant mutant can restore the integrity of mitochondrial cristae structure, correct disturbances in cellular redox homeostasis, and thereby effectively inhibit the malignant progression of liver cancer [14]. Wu and co-workers provided additional evidence linking Myo19 to the regulation of mitochondrial cristae. Loss of Myo19 increases mitochondrial ROS levels, which, in turn, contributes to enhanced tumor cell invasion and migration [15]. In addition to regulating ROS production, mitochondria play a key role in metabolic reprogramming. Mitochondrial dysfunction and increased ROS can exacerbate the Warburg effect characteristic of cancer cells, whereby glycolysis is preferentially maintained even under aerobic conditions [16]. Furthermore, one study reported that the Warburg effect is significantly enhanced in chronic myeloid leukemia (CML) cells that have acquired resistance to imatinib. The novel compound chiglitazar was shown to inhibit the Warburg effect through the PPARγ/mTOR/PKM2 pathway, thereby reducing glucose consumption and lactate production [17].

Overall, oxidative stress associated with abnormalities in mitochondrial membrane structure can not only directly promote tumor progression but also contribute to tumor development and drug resistance by altering metabolic reprogramming in tumor cells. In the context of glycolysis-targeted therapies, simultaneous targeting of mitochondrial ROS may therefore produce effects that differ from those anticipated from glycolytic inhibition alone.

#### 2.1.2. NADPH Oxidases (NOXs)

The NADPH oxidase (NOX/DUOX) family at the plasma membrane is a principal source of enzymatically produced ROS. Under the control of the small GTPase RAC and cytoplasmic PHOX cofactors, membrane-bound catalytic subunits (NOX1-5 and DUOX1/2) transfer electrons from NADPH to oxygen, yielding O_2_·^−^ (Figure 1). A landmark study indicates that once O_2_·^−^ has been converted into H_2_O_2_ by SODs, it diffuses into the microenvironment and induces oxidative stress in neighbouring cancer-associated fibroblasts (CAFs), driving their evolution towards a more invasive and immunosuppressive phenotype [18]. This phenomenon can drive sustained activation of key pro-survival and pro-proliferative pathways, among them MAPK, PI3K/AKT, NF-κB, and STAT3 [19]. Gorlach-Lira and colleagues demonstrated that excess H_2_O_2_ activates NF-κB (RelA/p65) in liver cancer cells, promoting APE1/Ref-1 accumulation and reinforcing cell survival [20]. Fine-tuning NOX activity therefore enables cancer cells to maintain redox balance, limiting oxidative damage while preserving ROS-dependent survival signaling. From this perspective, the NOX system is not merely a source of ROS but also functions as a signaling hub that integrates metabolic, stromal, and immune cues within the tumor microenvironment, a property that may inform the development of future redox-targeting strategies.

#### 2.1.3. Peroxisomes

Peroxisomes are small organelles in eukaryotic cells, each wrapped in a single membrane and highly conserved across species. Their core function is the production and degradation of H_2_O_2_. But in recent years they have been recognized as key hubs involved in lipid metabolism and the regulation of redox balance (Figure 1) [21]. With regard to the regulation of H_2_O_2_, peroxidoreductases (such as PRDX1, PRDX5 and CAT) in peroxisomes precisely regulate the homeostasis of low-concentration H_2_O_2_. They effectively prevent cytoplasmic oxidative damage caused by H_2_O_2_ leakage resulting from conformational changes in xanthine oxidoreductase (XOR), thereby playing a key role in maintaining cellular redox homeostasis [22]. However, in recent years, researchers have taken a keen interest in its role in regulating ROS through lipid metabolism. The peroxisomal acyl-CoA oxidase (ACOX) family (such as ACOX1, ACOX2) comprises key fatty acid oxidases in peroxisomes. Ibanez et al. demonstrated that ACOX1 promoted tumor growth and migration by enhancing ACOX1-mediated fatty acid oxidation (FAO) [23]. However, ACOX1-mediated FAO also generates H_2_O_2_ as a byproduct, which can exert growth-inhibitory effects under certain contexts. Furthermore, another study showed that inhibiting ACOX1 improves the survival of multiple myeloma cells under the stress of chemotherapeutic agents (such as bortezomib), which also highlights its role in drug resistance [24]. ACOX2, on the other hand, exhibits the opposite function. For example, Zhang et al. reported that its expression was sharply declined in hepatic carcinoma tissues [25]. This may indicate a low demand for branched-chain fatty acids in the metabolism of liver cancer cells. Interestingly, tumor cells, such as colonic cancer cells, also upregulate carnitine palmitoyltransferase 1A (CPT1A) to promote mitochondrial β-oxidation to obtain alternative energy sources and support cell membrane lipid synthesis, whilst abnormally inhibiting the activity of ACOX1 [25]. This regulatory strategy of compartmentalized metabolic reprogramming enables tumor cells to maximize the utilization of fatty acid catabolism for the production of adenosine triphosphate (ATP). Hussein and colleagues demonstrated that bypassing the growth-inhibitory H_2_O_2_ signal arising from ACOX1-mediated peroxisomal oxidation ultimately favors lipid storage and membrane synthesis, processes that are required for tumor progression [26]. Taken together, these findings imply a protective role for peroxisomes in cancer, one that operates through ROS and in which lipid metabolic regulation appears to be a particularly important element. The same observations raise the possibility that concurrent inhibition of peroxisomal FAO and mitochondrial β-oxidation could provide a viable approach to disrupting tumor metabolic plasticity.

#### 2.1.4. Endoplasmic Reticulum: The ERO1/PDI Oxidative Folding Machinery and Stress Adaptation

The endoplasmic reticulum (ER) is the central organelle responsible for protein synthesis, folding, and quality control in eukaryotic cells. It is also a prominent site of ROS production (Figure 1) [27]. During the maturation of secretory and membrane proteins, disulfide bond formation constitutes an energy-consuming oxidative folding process that generates ROS. This process is primarily catalyzed by the coordinated activities of the protein disulfide isomerase (PDI) family and ER oxidoreductase 1 (ERO1), predominantly ERO1α [27]. Researchers have increasingly focused on how the ER contributes to tumor development. Pan et al. found that when stimulated by the TME, frequent episodes of ER burden in cells, coupled with an abnormally elevated protein folding burden, significantly activate the ERO1α pathway, substantially amplifying ROS production [28]. In turn, activated ERO1α could promote tumor angiogenesis and metastasis [29]. At the same time, disrupted ER homeostasis may, via membrane contact sites, interact with mitochondrial dysfunction, thereby further exacerbating the accumulation of intracellular ROS, synergistically reshaping the tumor oxidative stress microenvironment, and contributing to the regulation of tumor malignant progression [30].

Overall, the ER contributes substantially to tumor development through its role in ROS production. In addition, its extensive membrane network provides an important interface linking ROS signaling to ferroptosis [30]. Therefore, further research into this area may also provide new approaches to cancer treatment.

#### 2.1.5. Exogenous ROS Generation

Exogenous ROS can arise from environmental pollution, ultraviolet (UV) radiation, chemical toxins, and ionizing radiation. Inhalation of air pollutants, industrial emissions, and vehicle exhaust, for instance, exposes the lungs to oxidizing agents and free radicals that promote ROS production [31]. Ultraviolet radiation, particularly UVA and UVB, triggers photochemical reactions in the skin, leading to ROS generation in epidermal cells. To date, UV radiation is an established environmental carcinogen, particularly for skin cancers, although its role in glioma development remains unclear [32]. Smoking, alcohol consumption, and exposure to certain drugs, pesticides, or heavy metals (e.g., lead, mercury, cadmium) act through similar mechanisms, activating oxidative enzymes or impairing mitochondria, to boost ROS accumulation [33]. Ionizing radiation, encompassing both gamma and X-rays, operates through a distinct mechanism: it directly splits water molecules, producing OH and other highly reactive species that can damage DNA and induce mutations (Figure 1) [34]. Such exogenous sources of oxidative stress may contribute substantially to carcinogenesis across a range of tumor types. Elucidating their molecular mechanisms and developing effective preventive strategies therefore remain important goals in cancer prevention.

### 2.2. ROS Resistance Strategies Adopted by Tumors

To withstand sustained oxidative stress during tumor progression, cancer cells establish a multilayered antioxidant network that maintains redox homeostasis. This adaptive system supports cell survival under elevated ROS levels and reduces susceptibility to therapeutic intervention. Key components of this network include the Nrf2–Keap1 axis, the glutathione/GPX4 system, the Trx/TrxR system, and NADPH-generating pathways. Across diverse tumor types, these systems play central roles in mounting effective responses to oxidative stress.

#### 2.2.1. Superoxide Dismutases (SODs)

SODs act as frontline antioxidant enzymes by catalyzing the dismutation of O_2_·^−^ to H_2_O_2_. In tumors, both SOD1 in the cytoplasm and SOD2 in mitochondria are highly expressed. Mitochondrial SOD2 is considered particularly critical for protecting cells from oxidative injury within that compartment [35]. This enzymatic activity has two major consequences: it helps preserve mitochondrial membrane integrity and limit mtDNA damage, while the resulting H_2_O_2_ can also serve as a substrate for redox signaling, for example, through the oxidation of protein thiols. Thus, beyond ROS detoxification, SODs also contribute to redox regulation in cancer cells (Figure 1). Upregulation of SOD2 restrains mitochondrial ROS accumulation and limits apoptosis under oxidative stress [36]. That said, the H_2_O_2_ produced can act as a diffusible messenger, activating MAPK, PI3K/AKT, and NF-κB cascades that support proliferation and adaptive remodeling [37]. SOD therefore may exert either protective or tumor-promoting effects depending on the redox environment. Tumor cells also increase ROS production through NOX family members, particularly NOX1, NOX2, and NOX4, creating a positive feedback loop with the SOD system [38]. O_2_·^−^ generated at the plasma membrane or within the ER is converted to H_2_O_2_ by SOD1 (or by extracellular SOD3), sustaining a stable redox signaling milieu [39]. Through coordinated regulation of NOX and SOD activities, tumors can maintain elevated basal ROS levels without exceeding the threshold for irreversible oxidative damage, a state often described as controlled oxidative stress. From a therapeutic perspective, this circuitry suggests that targeting ROS production or antioxidant capacity alone may be insufficient; effective disruption of tumor redox homeostasis will likely require combined strategies targeting multiple regulatory nodes.

#### 2.2.2. PRDX

Peroxiredoxins (PRDXs), a family of thioredoxin-dependent antioxidant enzymes, are widely distributed across diverse subcellular compartments. They efficiently reduce H_2_O_2_ to H_2_O using reducing equivalents supplied by thioredoxin (Trx), with NADPH serving as the ultimate electron donor through the Trx system (Figure 1). During the early stages of tumor development, PRDXs can exert tumor-suppressive effects by scavenging ROS, thereby preserving genomic stability and limiting the accumulation of mutations. Interestingly, a recent preprint study published on bioRxiv preliminarily reported that PRDX1-knockout mice exhibited genomic instability, reduced lifespan, and accelerated tumor development, including the emergence of malignant tumors such as lymphoma, sarcoma, and carcinoma [40]. However, as tumors progress, the potent ROS-scavenging activity of PRDXs can paradoxically become an important mechanism that allows tumor cells to evade oxidative damage and develop drug resistance. Wu and co-workers reported that multiple PRDX family members, including PRDX1, PRDX2, and PRDX6, are frequently upregulated across various cancers. Moreover, such upregulation is strongly associated with aggressive tumor behavior, resistance to chemotherapy, and immune evasion [41]. Therefore, targeting PRDXs or interfering with their regulatory axes (such as the Trx system) is considered a potential pathway to enhance sensitivity to ROS-related therapeutic strategies.

#### 2.2.3. GPXs

GPXs are a family of antioxidant enzymes that use GSH to reduce peroxides. By participating in ROS scavenging and the reduction of lipid peroxides, GPXs can protect cancer cells from oxidative damage and thereby influence cell death pathways, including necrosis and apoptosis (Figure 1) [42]. GPXs play a dual role in tumor development [43]. Carmen et al. found that GPX1 expression was consistently elevated during the enrichment of cancer stem cells (CSCs) in myxoid liposarcoma (MLS). Notably, GPX1 knockdown impaired CSC proliferation and reduced their tumor spheroid-forming capacity, while also significantly suppressing tumor formation in vivo [44]. In turn, an interesting study has found that, in human melanoma A375 cells, GPX1 is enlisted to the ER by OSBPL8, where it directly diminishes oxidized phosphatidic acid, thereby inhibiting ROS-driven lipid peroxidation and ferroptosis [45].

In the context of redox-based cancer therapy, the pro-tumorigenic side effects of GPXs arguably constitute one of the most critical obstacles to current practice [44]. Determining the context-specific roles of GPXs in individual tumors remains critical for assessing their therapeutic potential. Accordingly, patient-specific characterization of GPX activity and redox dependence may be an important component of future precision medicine strategies.

#### 2.2.4. Glutathione (GSH)

GSH stands as the primary non-enzymatic antioxidant, safeguarding redox balance within cells. GSH not only directly scavenges ROS such as H_2_O_2_ and OH, but also facilitates the activity of antioxidant enzymes like GPX and GST, participating in the clearance of lipid peroxides and electrophilic metabolic by-products (Figure 1) [31]. In the development of tumors, the synthesis rate of GSH is regarded as a key factor. The xCT transporter is an important transporter that supplies substrates for GSH synthesis. Lee et al. proposed that high xCT expression not only enhances antioxidant capacity but also maintains GPX4 activity, preventing lipid peroxide accumulation and acting as a critical barrier against ferroptosis [46]. Once tumor cells have achieved a sufficient GSH synthesis rate, it could help tumor survival positively. To illustrate, in a work by Peng et al. [47] on brain metastases in lung cancer, the Wnt/NR2F2 signaling axis was found to transcriptionally activate GPX4. The upregulation of GPX4 led to high GSH consumption, which, by inhibiting ferroptosis, rendered cancer cells resistant to platinum-based chemotherapeutic agents.

Targeting the GSH metabolic pathway, especially through tumor-selective inhibition of its unique regulatory nodes, is a promising and effective approach. Furthermore, a strategy aimed at restoring sensitivity to chemotherapeutic agents that disrupt the redox balance of tumor cells is undoubtedly feasible.

#### 2.2.5. NADPH

NADPH is a core molecule sustaining the antioxidant capacity of tumor cells [48]. By providing reducing equivalents to glutathione reductase (GR) and thioredoxin reductase (TrxR), NADPH maintains GSH and Trx in their reduced, active states, thereby supporting the function of antioxidant enzymes such as GPXs and PRDXs (Figure 1). A highly influential study reported that, under elevated oxidative stress, pancreatic cancer cells upregulate glucose-6-phosphate dehydrogenase (G6PD), thereby increasing the cellular pools of NADPH and NADP^+^ and promoting tumor cell survival and metastasis [49]. Furthermore, research by Ö. Canli et al. revealed that NADPH is also involved in the repair of oxidative DNA injury, the regulation of lipid metabolism and apoptotic signaling cascades, thereby enhancing the resistance of tumor cells to chemotherapeutic agents such as temozolomide (TMZ) [50]. Such effects demonstrate that NADPH is an indispensable component of tumors. Consequently, exploring cancer treatments by targeting it represents a viable strategy. Han et al. discovered that using the clinical-stage small-molecule drug RRx-001 to inhibit G6PD depletes NADPH. This leads to a redox imbalance, ultimately triggering a newly identified form of programmed cell death (PCD) termed ‘disulfidptosis’ [51]. Furthermore, by inhibiting fatty acid synthase (FAS), its substrate NADPH accumulates within the cell, thereby stimulating NOW to produce ROS, which ultimately elicits apoptosis in breast cancer cells [52]. Overall, NADPH contributes not only to tumor progression but also to the therapeutic response of tumor cells. This dual role, analogous to the context-dependent effects of ROS in cancer, underscores the importance of maintaining NADPH homeostasis. Disruption of this balance beyond the tumor cell’s adaptive capacity may therefore provide a potential therapeutic opportunity.

### 2.3. The Dual Role of Oxidative Stress in Cancer: Promoting and Suppressing Tumorigenesis

Oxidative stress is defined as a cellular and molecular state resulting from an imbalance between ROS generation and elimination. Within the classical framework of biology, ROS were traditionally regarded as byproducts of aerobic metabolism and were characterized primarily as harmful molecules because of their high reactivity and capacity to damage biomolecules through oxidation. However, accumulating evidence has established that ROS are not merely metabolic byproducts but also function as signaling molecules that respond dynamically to extracellular and intracellular stimuli. In cancer, oxidative stress exerts context-dependent and opposing effects: it can promote tumor growth and dissemination while also inducing cancer cell death when ROS levels exceed the cellular tolerance threshold. This duality reflects the highly regulated nature of ROS responses, which vary according to cell type, tumor stage, and the tumor microenvironment (TME).

#### 2.3.1. Carcinogenic Effects

Oxidative stress can influence cancer development at multiple stages, from early initiation through disease progression. ROS (particularly H_2_O_2_) can oxidize cysteine residues on target proteins, thereby modulating the activity of various signaling cascades [5]. Myeloid-derived H_2_O_2_, in particular, has emerged as a key player in tumorigenesis. Canli and colleagues demonstrated that H_2_O_2_ accumulation in myeloid-specific GPX4-knockout mice triggers ROS production and DNA mutations in neighboring epithelial cells, driving malignant transformation [50]. At the genomic level, ROS preferentially oxidize guanine bases and induce several forms of DNA damage, including oxidized base mispairing and DNA crosslinks. These lesions can promote replication errors and disrupt DNA helical structure, thereby compromising the fidelity of DNA repair. When such damage remains unrepaired, mutations and chromosomal abnormalities accumulate, shifting the balance toward carcinogenesis [53].

At the signaling level, sustained ROS activation of the MAPK/ERK, PI3K/AKT, and NF-κB pathways can enhance tumor cell proliferation, migration, and invasion. In parallel, ROS can impair apoptotic responses and thereby promote cancer cell survival [54]. ROS also inflict direct genomic damage—oxidizing DNA bases (e.g., generating 8-oxoguanine), inducing double-strand breaks, and altering chromatin architecture—while simultaneously impairing DNA repair mechanisms, resulting in genomic instability and increased mutational burden [55]. Beyond the cancer cell itself, ROS reshape the TME in ways that favor malignancy. A comprehensive analysis shows that sustained oxidative signaling recruits CAFs and TAMs, induces immunosuppressive factors, attracts regulatory T cells (Tregs), and dampens effector T cell activity, collectively fostering immune evasion [56]. ROS additionally drive angiogenesis, remodel the extracellular matrix (ECM), and promote epithelial–mesenchymal transition (EMT), all of which potentiate metastatic spread [57]. These points will be explored further in subsequent sections.

#### 2.3.2. Anticancer Effects

ROS can switch to a tumor-suppressive mode when confined to specific concentrations and spatiotemporal settings. If intracellular ROS levels outstrip the redox homeostasis threshold tolerable by cancer cells, they can directly induce oxidative damage accumulation and trigger cell death programs. High levels of H_2_O_2_ or O_2_·^−^ can oxidize mitochondrial membrane lipids and proteins, with mitochondrial depolarization, cytochrome c release, and caspase activation, release of cytochrome c, and activation of the caspase cascade, thereby initiating the intrinsic apoptosis pathway [58]. Excessive ROS can enhance pro-apoptotic signaling output by oxidatively modifying stress-related molecules such as ASK1, JNK, and p38 MAPK, thereby suppressing tumor cell survival [59]. At the genomic level, severe oxidative stress induces widespread DNA double-strand breaks and replication fork collapse, activating the ATM/ATR–CHK1/CHK2 axis and p53-dependent damage responses and consequently triggering cell-cycle arrest and senescence [60]. When DNA repair fails, cells may instead undergo apoptosis or other forms of programmed cell death. In contrast to the low-level ROS that can promote adaptive selection and tumor progression, acute or uncontrolled ROS surges can cause extensive genomic damage and thereby restrain tumor growth.

Beyond apoptosis, ROS can also suppress tumor growth by inducing ferroptosis, an iron-dependent form of cell death characterized by the accumulation of lipid peroxides and consequent loss of membrane integrity. When antioxidant systems such as GPX4 or the GSH pathway are impaired, ROS-driven lipid peroxidation is intensified, potentially preferentially eliminating tumor cells that have undergone extensive metabolic reprogramming [61]. Oxidative signals originating within the TME can also modulate immune responses. Under certain conditions, moderate ROS levels appear to facilitate dendritic cell maturation and antigen presentation, and may contribute to CD8^+^ T-cell effector function [62].

The relationship between ROS and tumor behavior thus appears to follow a hormetic pattern: relatively low to moderate ROS levels often favor tumor adaptation and progression, whereas once a critical threshold is exceeded, the same ROS can become cytotoxic and drive cells toward death.

## 3. Key Signaling Pathways of Oxidative Stress

Reversible oxidation of cysteine residues by ROS can alter the conformation, activity, or subcellular localization of signaling proteins. These modifications can modulate downstream signaling by either enhancing or attenuating pathway activity. Such redox regulation influences cell proliferation, apoptosis, metabolic flux, and immune responses and, in tumors, may contribute to malignant transformation, disease progression, and therapeutic resistance.

### 3.1. Nrf2-Keap1 Pathway and ROS

#### 3.1.1. Overview of the Nrf2 Pathway

**Figure 2:**
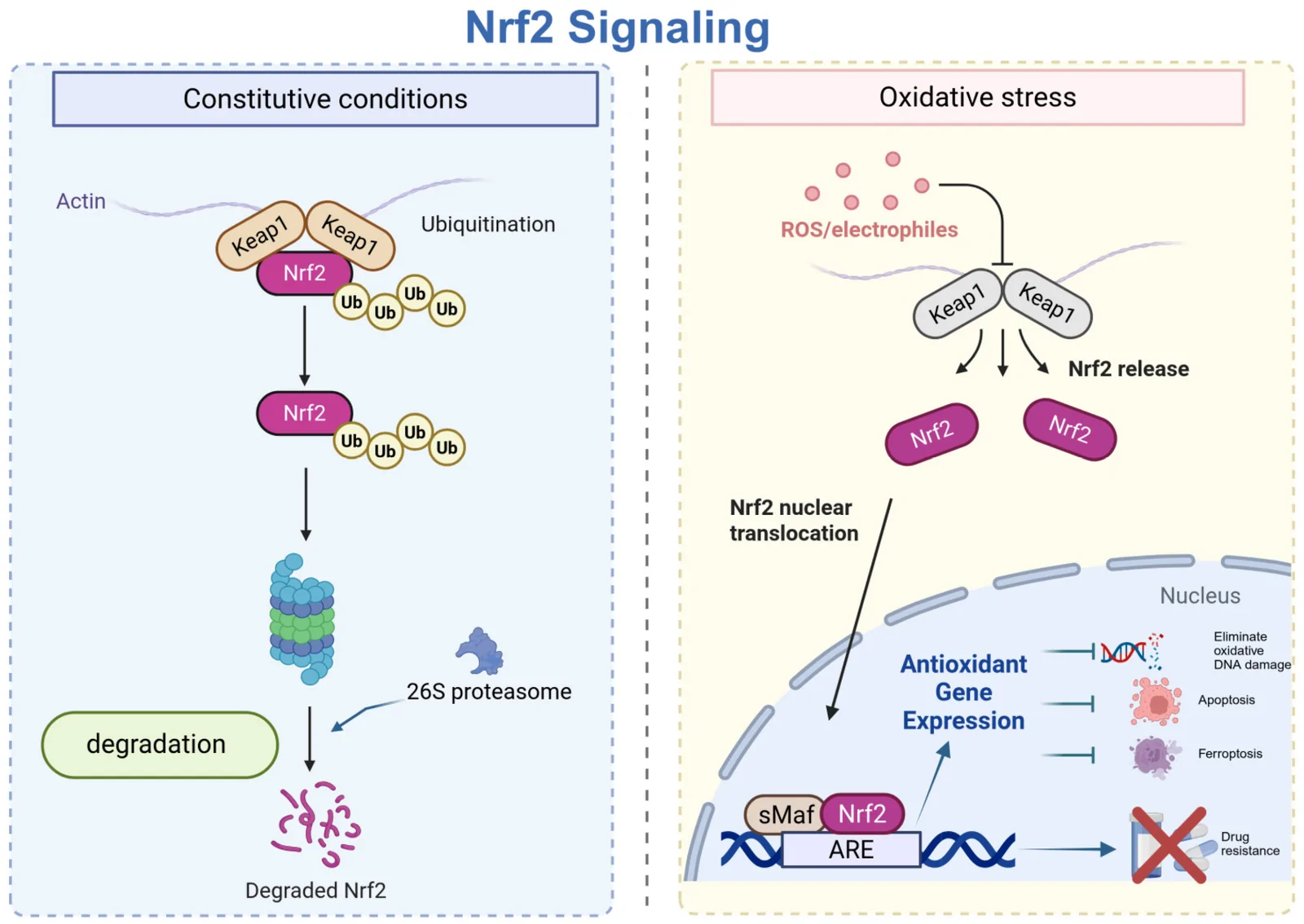
Nrf2–Keap1 signaling axis in the regulation of cellular redox homeostasis. Basal state (**left**): Under basal conditions, Keap1 dimers continuously promote Nrf2 ubiquitination through the Cullin 3 (Cul3)-E3 ubiquitin ligase complex, in which Keap1 functions as the substrate-recognition subunit. Polyubiquitinated Nrf2 is subsequently targeted for proteasomal degradation, resulting in low steady-state Nrf2 protein levels. Oxidative/electrophilic stress (**right**): Upon accumulation of ROS or electrophilic species, reactive cysteine residues in Keap1, including Cys151, undergo oxidative or covalent modification. These modifications impair Keap1-mediated ubiquitination of Nrf2, allowing newly synthesized Nrf2 to accumulate and translocate to the nucleus. In the nucleus, Nrf2 heterodimerizes with small Maf proteins and binds to antioxidant response elements (AREs) in the promoter regions of target genes, including HMOX1, NQO1, and GCLC. The resulting transcriptional response enhances antioxidant gene expression and promotes restoration of redox homeostasis. In many tumor contexts, this adaptive mechanism may confer increased resistance to oxidative stress associated with therapeutic intervention.

Nrf2 is a CNC-bZIP transcription factor that plays a central role in maintaining redox homeostasis by orchestrating the expression of antioxidant genes. Through ARE-dependent transcription, Nrf2 regulates a broad range of downstream processes, including antioxidant defense, detoxification, and cellular adaptation to stress [63]. Structurally, Nrf2 contains several conserved Neh domains. Among these, Neh1 is particularly important because it contains the CNC-bZIP region required for DNA binding and heterodimerization with small Maf proteins, whereas the Neh2 domain mediates the interaction of Nrf2 with Keap1 (Figure 2) [64]. Under steady-state conditions, Nrf2 binds to Keap1 via its Neh2 domain and is recruited to the Cullin3 (Cul3)-E3 ubiquitin ligase complex, undergoing continuous ubiquitination and subsequent proteasomal degradation [65], thereby maintaining low expression levels. When cells are exposed to ROS or electro-affinity stress molecules, key cysteine residues of Keap1 undergo oxidation or covalent modification, inducing conformational alterations in and diminution of its inhibitory function on Nrf2. Undegraded Nrf2 hangs out in the cytoplasm until it gets the signal to head into the nucleus, where it forms heterodimers with small Maf proteins. This complex binds to the ARE sequence, initiating the expression of multiple antioxidant and metabolism-related genes [66]. Beyond classical Keap1-dependent regulation, Nrf2 can also be activated through non-canonical pathways. For instance, p62/SQSTM1 accumulates during impaired autophagy and competitively binds Keap1, blocking its ubiquitination of Nrf2 [67].

#### 3.1.2. Dynamic Equilibrium Regulation of the Nrf2 Pathway and ROS

The Nrf2-Keap1 axis serves as a primary sensor of ROS and coordinates downstream adaptive responses. Under moderate oxidative conditions, Nrf2 activation drives glutathione synthesis. It can support NADPH regeneration and facilitate peroxide elimination; all of these contribute to restoring redox balance. An alternative mode of regulation was reported by Zhou and colleagues: baicalin increases Keap1 expression, which promotes Nrf2 ubiquitination and proteasomal breakdown. The result is diminished Nrf2 levels and reduced expression of targets including GPX4 and SLC7A11, and under certain conditions may precipitate ferroptosis in tumor cells [68]. These findings further support the role of Nrf2 in protecting cells against oxidative stress.

In established tumors, sustained Nrf2 activity frequently drives redox reprogramming. A well-known study in pancreatic ductal adenocarcinoma (PDAC) demonstrated that Nrf2 induces *EZH2* transcription, while EZH2 in turn co-activates *NFE2L2*, the gene encoding Nrf2, thereby generating a positive feedback loop that reinforces redox adaptation in PDAC [69]. In this loop, antioxidant and detoxification genes are upregulated, which increases tolerance to high ROS levels. Nrf2 also influences metabolic pathway choice. For instance, it upregulates enzymes of the pentose phosphate pathway (PPP) and serine synthesis, thereby enhancing NADPH production for reductive reactions [70].

Beyond its role in ROS detoxification, Nrf2 contributes to metabolic adaptation and biosynthetic processes in tumor cells. Its activity does not simply reduce ROS concentrations; rather, it appears to maintain ROS within a range that favors cancer cell survival and proliferation—sufficient to sustain growth-promoting signals while remaining below the threshold that induces lethal oxidative damage. This calibrated regulation may allow tumor cells to sustain high metabolic activity while limiting oxidative injury.

#### 3.1.3. Role of the Nrf2 Pathway in Tumorigenesis and Progression

In the early phases of tumorigenesis, Nrf2 may exert tumor-suppressing functions. One such mechanism involves the upregulation of reducing agents, which facilitates the clearance of oxidative DNA damage. In addition, Nrf2 can inhibit lipid peroxidation and contribute to the maintenance of genomic stability, thereby supporting its protective role in this context. However, following tumor formation, numerous reports have demonstrated a phenomenon contrary to that observed in the early phases of tumor development. Tumor cells can prolong overactivation, which is frequently exploited by cancer cells. For instance, Chang et al. found that, in ovarian cancer, Cancer cells showed much stronger migration and invasion when Nrf2 was elevated [71]. In addition, they also reported that overexpression of Nrf2 significantly enhanced the resistance of tumor stem cell-like cells to paclitaxel and carboplatin [71]. Nrf2 also interacts with the tumor microenvironment (TME). Within tumors, treatment-induced stress or necrosis can activate Nrf2 in tumor-associated macrophages (TAMs), promoting their transition toward a stress-induced TAM phenotype. These TAMs suppress immune-activating pathways such as IFN–STAT1 signaling and exhibit reduced antigen-presenting and T-cell-recruiting capacities, thereby promoting tumor invasion and metastasis [72].

Collectively, the Nrf2 pathway exhibits context-dependent and dual functions in tumor biology. In normal tissues and during early tumorigenesis, Nrf2 maintains redox homeostasis and provides an antimutagenic defense against oxidative damage. In established tumors, however, Nrf2 is frequently hijacked to support antioxidant defense, metabolic adaptation, and therapeutic resistance. Genetic or functional dysregulation of the Keap1–Nrf2 axis is observed across multiple tumor types and represents an important target for redox-based therapeutic strategies.

### 3.2. NF-κB Pathway and ROS

#### 3.2.1. Overview of the NF-κB Pathway

NF-κB (nuclear factor-kappa B) comprises a highly conserved family of transcription factors, including RelA (p65), RelB, c-Rel, p50 (NF-κB1), and p52 (NF-κB2). Through the formation of homodimers or heterodimers, these proteins regulate diverse cellular processes, including cell proliferation, inflammatory responses, immune regulation, and resistance to apoptosis [73]. All NF-κB family members contain a Rel homology domain (RHD) that mediates DNA binding, dimerization, and interaction with IκB proteins. Under basal conditions, IκB proteins, particularly IκBα, sequester NF-κB complexes in the cytoplasm and maintain them in an inactive state. NF-κB signaling can be activated through two major pathways: the well-characterized canonical pathway and the non-canonical pathway.

**Figure 3:**
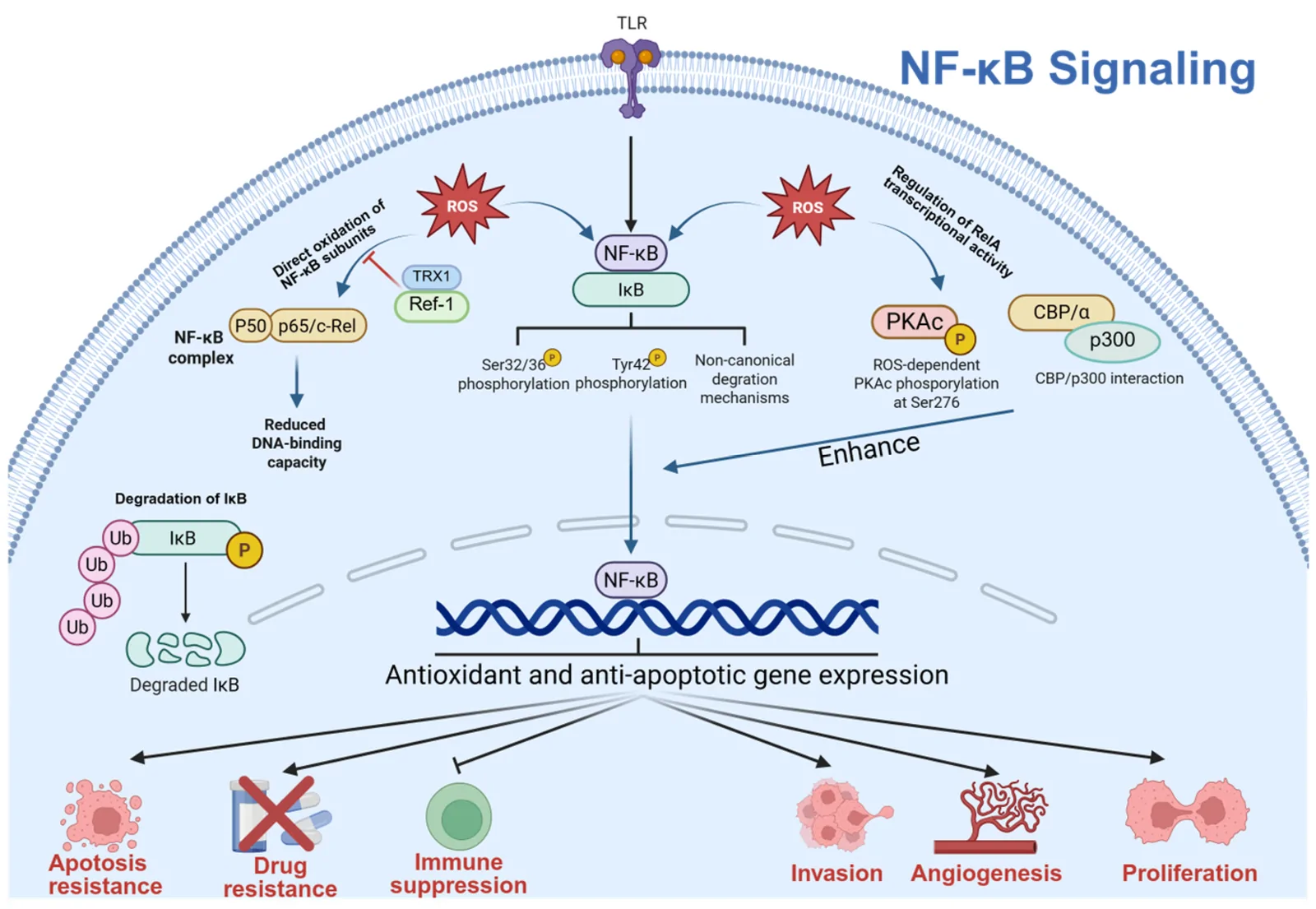
ROS-mediated bidirectional regulation of the NF-κB signaling pathway and its downstream cellular effects. Toll-like receptor (TLR) activation triggers the NF-κB cascade, in which IκB phosphorylation at Ser32/36 promotes its ubiquitination and proteasomal degradation. Upon IκB breakdown, bound NF-κB dimers (p50/p65/c-Rel) are liberated, translocate into the nucleus, and initiate subsequent transcriptional programs. ROS modulate this pathway at multiple levels. On one hand, ROS directly oxidize NF-κB subunits (e.g., p50 at Cys-62), impairing DNA-binding activity, a modification that can be reversed by the Ref-1/TRX1 redox system. On the other hand, ROS regulate upstream and transcriptional events, including non-canonical phosphorylation of IκB (e.g., Tyr42) and ROS-dependent PKAc-mediated phosphorylation of RelA at Ser276, which enhances its interaction with coactivators CBP/p300 and promotes transcriptional activity. NF-κB drives antioxidant and survival gene expression, promoting therapy resistance, immune escape, and malignant progression.

The canonical pathway is activated by pro-inflammatory cytokines, pathogen-associated molecular patterns such as lipopolysaccharide (LPS), or antigen receptor signaling. These stimuli activate the IKK complex, which phosphorylates IκB proteins and targets them for ubiquitin-dependent degradation. Following IκB degradation, NF-κB subunits, typically p50/RelA, translocate to the nucleus and activate the transcription of target genes (Figure 3) [74]. The alternative NF-κB pathway primarily activates NF-κB-inducing kinase (NIK) through receptor signaling (e.g., lymphotoxin β receptor, CD40, BAFF-R), selectively activating the IKKα homodimer. This promotes processing of the p100 precursor protein into p52, which ultimately forms the p52/RelB complex for transcriptional activation [75].

#### 3.2.2. Interactions Between the NF-κB Pathway and ROS

To counteract ROS-induced toxicity, NF-κB signaling can respond to oxidative stress by inducing the expression of multiple anti-apoptotic and antioxidant genes, thereby contributing to redox homeostasis and influencing the fate of cancer cells. ROS can also indirectly activate the NF-κB signaling cascade. A widely cited study reported that, during the progression of HCC, accumulated ROS induce oxidative modification of Pirin, a co-activator protein, in the cytoplasm and promote its translocation to the nucleus. Pirin enhances NF-κB transcriptional activity, leading to increased production of TNF-α and IL-6 [76]. These cytokines, in turn, promote intracellular ROS generation, establishing a positive feedback loop that may further accelerate HCC progression [76]. These cytokines, in turn, promote the generation of intracellular ROS; this positive feedback can accelerate the progression of HCC [76].

ROS can also directly modulate NF-κB dimers. Direct ROS oxidation of NF-κB typically occurs at the Cys62 residue of its p50 through S-glutathionylation. This leads to a reduction in the binding capacity of NF-κB to DNA [77]. However, direct interaction between the intracellular ribonucleoprotein Ref-1 (APE1), involved in base excision repair, and TRX1 may selectively reduce this oxidation [78], thereby restoring NF-κB’s DNA-binding capacity. Additionally, ROS can regulate upstream components of the NF-κB signaling. Exogenous H_2_O_2_ modulates NF-κB activity through non-canonical mechanisms. Under normal conditions, IKK-mediated phosphorylation of IκBα at Ser32/36 marks it for ubiquitination and subsequent degradation, thereby releasing NF-κB. In contrast, H_2_O_2_ induces phosphorylation of tyrosine residues such as Tyr42 on IκBα. Although this post-translational modification does not always induce degradation, it effectively tunes NF-κB dissociation and downstream activation [79].

#### 3.2.3. Effects of NF-κB Pathway Activation on Tumors

During tumorigenesis, the tumor-promoting role of NF-κB signaling is supported by substantial evidence. The NF-κB pathway contributes to genomic instability by promoting the production of ROS and reactive nitrogen species (RNS), while simultaneously inducing the expression of anti-apoptotic and cell-cycle regulatory proteins. NF-κB transcriptionally upregulates multiple anti-apoptotic proteins, including inhibitor of apoptosis proteins (IAPs) such as c-IAP1, c-IAP2, XIAP, and c-FLIP. These factors inhibit caspase activation and, in particular, can interfere with caspase-8-dependent signaling, thereby attenuating extrinsic apoptotic signaling and promoting cancer cell survival [80,81]. ROS-activated NF-κB also promotes tumor progression by regulating genes governing EMT and ECM remodeling, thereby enhancing metastasis. A growing body of evidence, including a recent study [82], points to the ROS/NF-κB/MMP-9 regulatory axis as a major contributor to colorectal cancer progression, particularly in terms of invasion and metastasis. Additionally, NF-κB appears to facilitate immune suppression and fibroblast-mediated ECM remodeling within the TME, and these effects, taken together, seem to promote the long-term survival of malignant cells. Furthermore, within the activated TME, NF-κB fosters immune suppression and ECM remodeling by fibroblasts, both of which support cancer cell survival. For instance, in colorectal cancer, tumor cells recruit neutrophils (PMNs) and upregulate PD-L1 on their surface by activating the NF-κB/CXCL1 axis. This induces CD8^+^ T cells to enter a state of exhaustion, thereby losing their ability to kill tumor cells [83]. Overall, NF-κB signaling exerts a central regulatory role in tumors through coordinated interactions across multiple pathways and cellular compartments. Sustained NF-κB activation not only supports tumor cell proliferation, survival, and self-renewal but also contributes to therapeutic resistance through the regulation of oxidative stress, cellular metabolism, and the immune microenvironment. The canonical and non-canonical NF-κB pathways together constitute an important regulatory network in tumor biology and represent potential therapeutic targets. Further clarification of how NF-κB-regulated programs interact with the TME may facilitate the development of more precise and context-specific therapeutic strategies.

### 3.3. MAPK Pathway: ROS-Mediated Proliferation and Apoptosis Signaling

#### 3.3.1. Overview of the MAPK Pathway

In mammalian cells, the MAPK family comprises three major branches: ERK, JNK, and p38. These kinase cascades mediate distinct but partially overlapping cellular responses. ERK primarily transduces mitogenic signals, whereas JNK and p38 are predominantly activated by cellular stress and inflammatory stimuli, respectively [84]. Signal transduction through MAPKs proceeds via a three-tier phosphorylation cascade (MAPKK kinase kinase (MAPKKK) → MAPK kinase (MAPKK) → Mitogen-activated protein kinase (MAPK)), relaying external cues to the nucleus with precision. Mitogen-activated protein kinase phosphatases (MKPs) keep this system tightly controlled.

**Figure 4:**
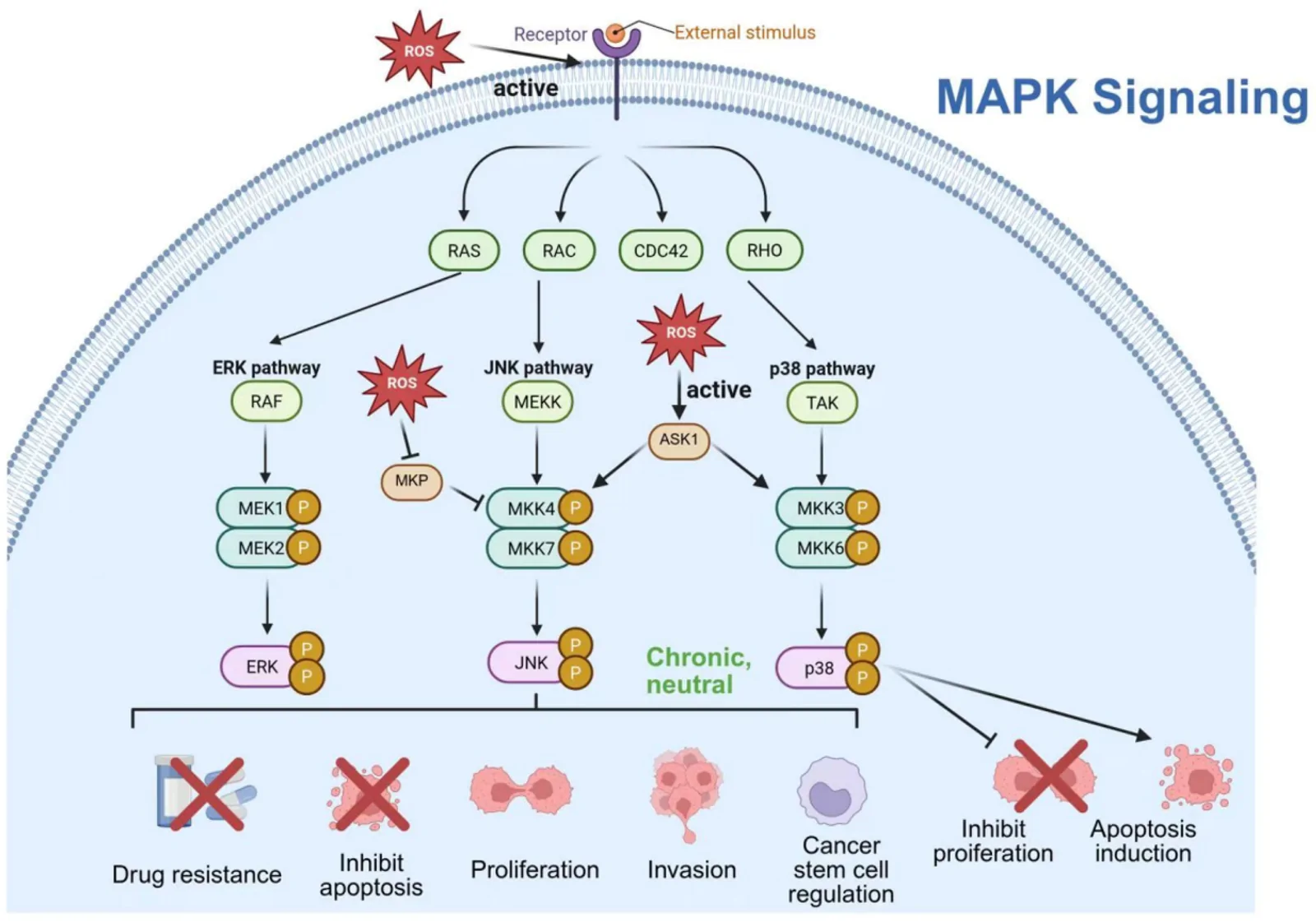
The three principal MAPK cascades, RAF-MEK-ERK, MEKK-MKK4/7-JNK, and TAK-MKK3/6-p38, are typically triggered when extracellular signals engage membrane receptors and small GTPases such as RAS, RAC, CDC42, and RHO. ROS can impinge on these pathways at multiple levels. For ERK, modulation may occur through receptor-dependent routes and upstream kinases, with signal strength further shaped by redox-sensitive intermediates. The JNK and p38 arms, in turn, are often activated under oxidative conditions; this involves, at least in part, the dissociation of ASK1 from reduced thioredoxin, followed by its oligomerization and downstream signaling. ROS also prolong pathway activation by impairing MKPs; oxidation of their catalytic cysteines reduces their ability to dephosphorylate MAPKs. The net effect under persistent oxidative stress is extended MAPK signaling, which tends to support proliferation, invasion, stem-like properties, and resistance to apoptosis and therapy. That said, stress-responsive branches like p38 can sometimes exert opposing effects. In certain settings, they can limit growth or even promote cell death, depending on the cellular context.

Extracellular signals such as growth factors, cytokines, or stress stimuli typically engage MAPKKK, which initiates a phosphorylation cascade through MAPKK to MAPK. Upon phosphorylation, MAPKs enter the nucleus and modulate transcription factors, cytoskeletal regulators, and other downstream effectors, thereby influencing proliferation, differentiation, migration, stress responses, and apoptosis [85]. The MAPK pathway is not restricted to stress signaling; it also conveys mitogenic signals. In many tumor contexts, aberrant MAPK activation may contribute to tumor initiation, progression, and therapy resistance. The interplay between ROS and MAPK continues to be explored; how ROS might modulate MAPK activity and the implications for cancer cell biology are discussed later in this review (Figure 4).

#### 3.3.2. Interactions Between ROS and the MAPK Pathway in Tumor

Dysregulated MAPK signaling is frequently implicated in tumor cell proliferation, survival, and therapeutic resistance. Among the three major MAPK branches, the RAS/RAF/MEK/ERK axis has been extensively studied. Aberrant activation of this cascade, particularly sustained ERK1/2 activity, is one of the most common MAPK alterations observed in human malignancies [86]. Current evidence suggests that ROS can modulate MAPK signaling through at least two distinct mechanisms. First, oxidative modification of MAPKKKs, which function at the upstream tier of the cascade, can promote their activation. Second, oxidative inactivation of MAPK phosphatases (MKPs), key negative regulators of MAPK signaling, can delay signal termination and thereby prolong downstream pathway activity.

Matsushita et al. found that ROS regulate MAPK signaling through MTK1. Under oxidative stress, evolutionarily conserved cysteine residues within MTK1 are rapidly oxidized, and this reversible redox modification activates its kinase activity, thereby triggering downstream MAPK signaling [87]. Turner-Ivey et al. reported that peroxidoreductase 1 (Prdx1) acts as a sensor that binds to MKP-1 and MKP-5. In the presence of high concentrations of H_2_O_2_, Prdx1 is excessively oxidized and dissociates from MKP-1, leading to oxidation-induced oligomerization and inactivation of MKP-1. Consequently, MKP-1 loses its ability to dephosphorylate p38MAPK α, thereby inhibiting the MAPK pathway [88]. Taking this experimental evidence together, ROS activate the MAPK pathway via two main mechanisms: directly through oxidative modification, which activates upstream MAPKKKs such as MTK1 and ASK1, and, alternatively, through oxidative modification, they inhibit the activity of negative regulatory proteins (like MKPs) such as MKP-1 and MKP-3. These two mechanisms act synergistically to ensure that oxidative stress signals can efficiently and sustainably activate the downstream MAPK pathway. They also ensure that tumor cells can freely regulate the MAPK pathway in their unique way via ROS.

#### 3.3.3. Effects of MAPK Pathway Activation on Cancer

Through these pathways, cells integrate diverse external and internal inputs to orchestrate proliferation, differentiation, survival, and migration [89]. This regulatory capacity places the MAPK network at the center of tumor development and progression, where it shapes malignant phenotypes and influences therapeutic responses and drug resistance. Among the three major MAPK branches, ERK, JNK, and p38 are well-established regulators of cancer cell proliferation, survival, and apoptosis. In particular, dysregulation of the RAS–RAF–MEK–ERK pathway contributes substantially to human tumorigenesis. Aberrant activation of this pathway not only promotes tumor cell proliferation, survival, invasion, and metastasis but also stimulates extracellular matrix (ECM) remodeling and angiogenesis, thereby creating a microenvironment conducive to tumor progression.

Within the MAPK/ERK pathway, Ma and colleagues reported that MARK4 activates MAPK/ERK signaling. siRNA-mediated knockdown of MARK4 suppressed gastric cancer cell proliferation, migration, and invasion [90]. In osteosarcoma cells (OS), Mukherjee et al. found that cisplatin, by inducing the accumulation of ROS, activates both the JNK pathway and autophagy; the crosstalk between these two mechanisms collectively contributes to the survival of osteosarcoma cells [91]. Indeed, the activation of p38 MAPK has been viewed in a contrasting light in triple-negative breast cancer (TNBC). In patient-originated organoid (PDO) models of TNBC, tumor growth is curbed by Toyocamycin, a natural compound that acts through p38 MAPK activation [92]. This may also explain how ROS activates p38 MAPK to inhibit tumor growth. In summary, members of the MAPK family exert synergistic effects on tumor cells through multiple mechanisms; their activation levels can both promote tumor progression and exert tumor-suppressing effects. Further elucidation of the mechanisms underlying the interaction between ROS and the MAPK signaling pathway is expected to provide new insights into the progression of novel anticancer therapies, the improvement of treatment tolerability for patients, and the targeted elimination of tumor stem cells.

### 3.4. PI3K/AKT/mTOR Pathway

#### 3.4.1. Overview of the PI3K/AKT/mTOR Pathway

**Figure 5:**
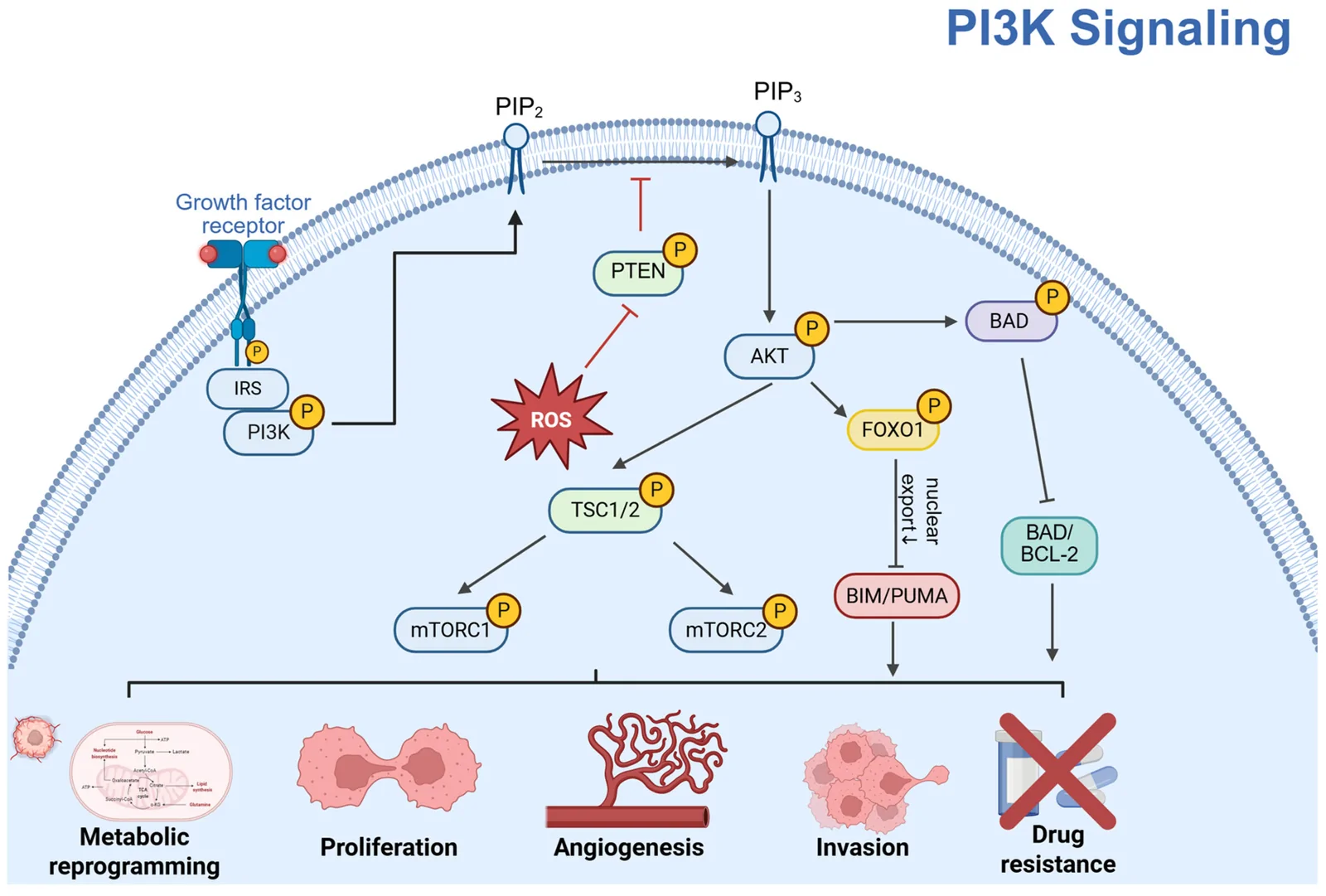
Upon activation of growth factor receptors, PI3K signaling is initiated through the conversion of PIP2 to PIP3, leading to AKT activation and subsequent engagement of mTORC1 and mTORC2. Activated AKT also phosphorylates BAD at Ser136, preventing its interaction with BCL-2/BCL-xL, and phosphorylates FOXO1, promoting its nuclear exclusion and suppressing the transcription of pro-apoptotic genes such as BIM and PUMA, thereby contributing to apoptosis resistance. ROS primarily modulate this pathway through effects on negative regulatory components. PTEN serves as a notable example: oxidation of its cysteine residues to Cys124 promotes disulfide bond formation and suppresses its phosphatase activity, which in turn allows PIP3 accumulation and sustained signaling. ROS can also inactivate protein tyrosine phosphatases (PTPs), further enhancing upstream phosphorylation events. Although thioredoxin and GSH-based systems can reverse these oxidative modifications, the persistently high oxidative environment typical of many tumors may favor pathway activation. Functional consequences associated with sustained PI3K/AKT/mTOR signaling include enhanced metabolic reprogramming, increased proliferation and angiogenesis, heightened invasion, therapy resistance, and apoptosis resistance.

PI3K signaling is a central regulator of cellular homeostasis, controlling cell growth, differentiation, metabolic adaptation, and survival. Activation of the pathway is typically initiated by receptor tyrosine kinases (RTKs), G protein-coupled receptors (GPCRs), or Ras, which stimulate the conversion of phosphatidylinositol 4,5-bisphosphate (PIP2) to phosphatidylinositol 3,4,5-trisphosphate (PIP3). This signaling event is negatively regulated by PTEN, a phosphatase that dephosphorylates PIP3 to regenerate PIP2. The accumulation of PIP3 recruits PDK1 and AKT to the plasma membrane, facilitating AKT phosphorylation and activation. Activated AKT inhibits pro-apoptotic signaling, promotes cyclin expression, and reprograms cellular metabolism, thereby supporting tumor cell growth and survival [93,94]. Downstream, mTOR assembles into two distinct complexes (mTORC1 and mTORC2), each with its own tasks [95]. mTORC1 drives translation and protein synthesis by phosphorylating 4E-BP1 and S6K1; mTORC2 sustains AKT activity through Ser473 phosphorylation, influencing cytoskeletal organization and metabolism (Figure 5) [96]. Hyperactivation of this axis is frequently implicated in driving unchecked proliferation, evasion of apoptosis, and metabolic reprogramming in cancer [97]. The pathway thus represents a potential target for anticancer therapy, and may also offer opportunities to counteract resistance and improve clinical outcomes.

#### 3.4.2. Crosstalk Between the PI3K/AKT/mTOR Pathway and ROS

The pathway and ROS can engage in a two-way crosstalk: ROS activate the pathway, and the pathway, in turn, shapes ROS production and clearance; together, they influence tumor cell growth and survival [98]. The Cys124 residue in PTEN is a key ROS target (Figure 5), and H_2_O_2_ generated by NOX, nitric oxide synthase (NOS), SOD on the cell membrane, or exogenous ROS oxidizes this site, forming an intermolecular disulfide bond with adjacent Cys71 [99]. This modification inactivates PTEN and consequently relieves its negative regulation of the PI3K/AKT/mTOR axis, resulting in pathway activation. In addition, an earlier study showed that ONOO^−^ can oxidatively modify cysteine residues in protein tyrosine phosphatases (PTPs), leading to their inactivation. At low concentrations, ONOO^−^ may induce PTP oxidation more efficiently than H_2_O_2_, thereby enabling rapid and effective inhibition of PTP activity [100]. Then, ONOO ^−^ is thus considered a major contributor to PTP inactivation, driving elevated tyrosine phosphorylation under oxidative stress. This modification is not permanent, however. Antioxidant systems, including the TRX system, GSH, GR, and peroxidases, can reduce the disulfide bond and restore PTEN function [101]. In cancer cells, persistent oxidative stress may overwhelm these antioxidant defenses, delay PTEN recovery, and thereby sustain PI3K pathway activation, ultimately promoting tumor cell proliferation and progression.

Interestingly, PTEN activity also limits neuroregeneration following neuronal injury, despite the importance of PI3K signaling in neural repair [102]. During repair, neurons have been found to generate ROS after taking up NOX2-containing exosomes released by macrophages. These ROS, by oxidizing PTEN, are able to activate the PI3K cascade, and this activation subsequently contributes to enhanced nerve regenerative capacity [103]. Based on this, it is speculated that the microenvironment surrounding glioblastoma multiforme (GBM) contains primed macrophages, which may employ a similar mechanism to produce exosomes that cause sustained activation of the PI3K pathway in GBM cells. Similarly, Kwon et al. indicated that growth factor stimulation, like by EGF, PDGF, or insulin, activated NOXs and oxidized PTEN, forming H_2_O_2_ that enhances PI3K/AKT signaling [104]. Growth factors prevalent in the TME play an active role in this process, suggesting another potential mechanism for PI3K pathway activation.

#### 3.4.3. Effects of PI3K/AKT/mTOR on Tumors

Among the most frequently dysregulated pathways in human cancers is the PI3K/AKT/mTOR cascade, whose aberrant activation fuels tumor progression across multiple histological types. PIK3CA is frequently activated by mutations in breast and colorectal cancers [105], along with PTEN loss in melanoma and GBM, which leads to sustained PI3K signaling enhancement, thereby promoting abnormal activation of AKT and mTOR [106]. Activated AKT suppresses apoptosis by phosphorylating pro-apoptotic proteins such as BAD and FOXO, while also promoting the stability or activity of anti-apoptotic factors. Specifically, AKT phosphorylates BAD at Ser136 and FOXO1, thereby inhibiting BAD-mediated apoptosis and reducing FOXO1-dependent transcription of pro-apoptotic genes such as BIM and PUMA (Figure 5). Studies further indicate that PI3K/AKT signaling promotes tumor angiogenesis by stabilizing hypoxia-inducible factor 1α (HIF-1α) and increasing vascular endothelial growth factor (VEGF) expression, thereby supporting tumor nutrient supply and metastatic potential [107]. Furthermore, AKT activation induces EMT, characterized by downregulation of E-cadherin and upregulation of vimentin [108], thereby enhancing cancer cell migration and infiltration capabilities. Notably, abnormal signaling in this pathway not only directly reduces cellular sensitivity to chemotherapy-induced apoptosis but also further enhances cancer cell survival by modulating DNA damage responses (DDR) and autophagy.

Overall, the PI3K/AKT/mTOR pathway is a central regulator of tumor cell survival, metabolism, invasion, and metastasis. ROS-mediated oxidation of PTEN may sustain PI3K/AKT/mTOR signaling, highlighting the close interplay between oxidative stress and this pathway during tumor progression. This interaction may provide additional opportunities for therapeutic intervention.

## 4. Interactions Between Oxidative Stress and the TME

Within the TME, ROS are generated by tumor cells, stromal cells, and infiltrating immune cells. These sources collectively establish a dynamic redox environment that undergoes continuous fluctuation. Importantly, ROS are not merely byproducts of cellular metabolism but can also function as intercellular signaling molecules that mediate communication between malignant and non-malignant cells. Through such signaling, ROS can modulate cellular metabolism, remodel the extracellular matrix (ECM), influence angiogenesis, and alter immune function. However, persistent or excessive ROS accumulation may also contribute to DNA damage, chronic inflammation, immune evasion, and therapeutic resistance, thereby facilitating tumor progression.

A notable feature of the TME is the ability of tumor cells to adapt to and exploit oxidative conditions rather than simply endure them. By modulating redox states both intracellularly and extracellularly, tumor cells can establish a redox environment that supports growth while remaining below the threshold for lethal oxidative damage. The following sections examine how ROS regulate intercellular communication and metabolic reprogramming within the TME and explore how these processes may collectively contribute to tumor growth, invasion, immune evasion, and therapeutic resistance.

### 4.1. Redox-Dependent Communication Between Tumor Cells and Stromal Compartments

Mitochondrial electron leakage, NOX activity, hypoxia, vesicle-borne oxidants, and extracellular matrix (ECM)-associated reactions represent several mechanisms that contribute to ROS generation within the TME. The TME comprises a heterogeneous network of tumor cells, immune cells, stromal populations, and ECM components operating within a dynamic microenvironment. The resulting oxidative milieu can, in many contexts, promote metabolic reprogramming, stromal activation, and impaired immune function.

#### 4.1.1. ROS-Driven Activation and Pro-Tumor Functions of CAFs

Among stromal cell populations, CAFs often exhibit heightened sensitivity to ROS. The activation of quiescent tissue fibroblasts in the TME may involve tumor-derived H_2_O_2_, together with soluble cues such as TGF-β, PDGF, and the chemokine CXCL12/SDF-1. These signals can act in concert to promote CAF activation. ROS-dependent signaling enhances SMAD activation, induces α-smooth muscle actin (α-SMA) expression, and promotes acquisition of a myofibroblastic CAF phenotype [109]. ROS also drive monocyte-to-myofibroblast transdifferentiation through p38 mitogen-activated protein kinase (p38 MAPK) signaling, further expanding the CAF compartment [109].

**Figure 6:**
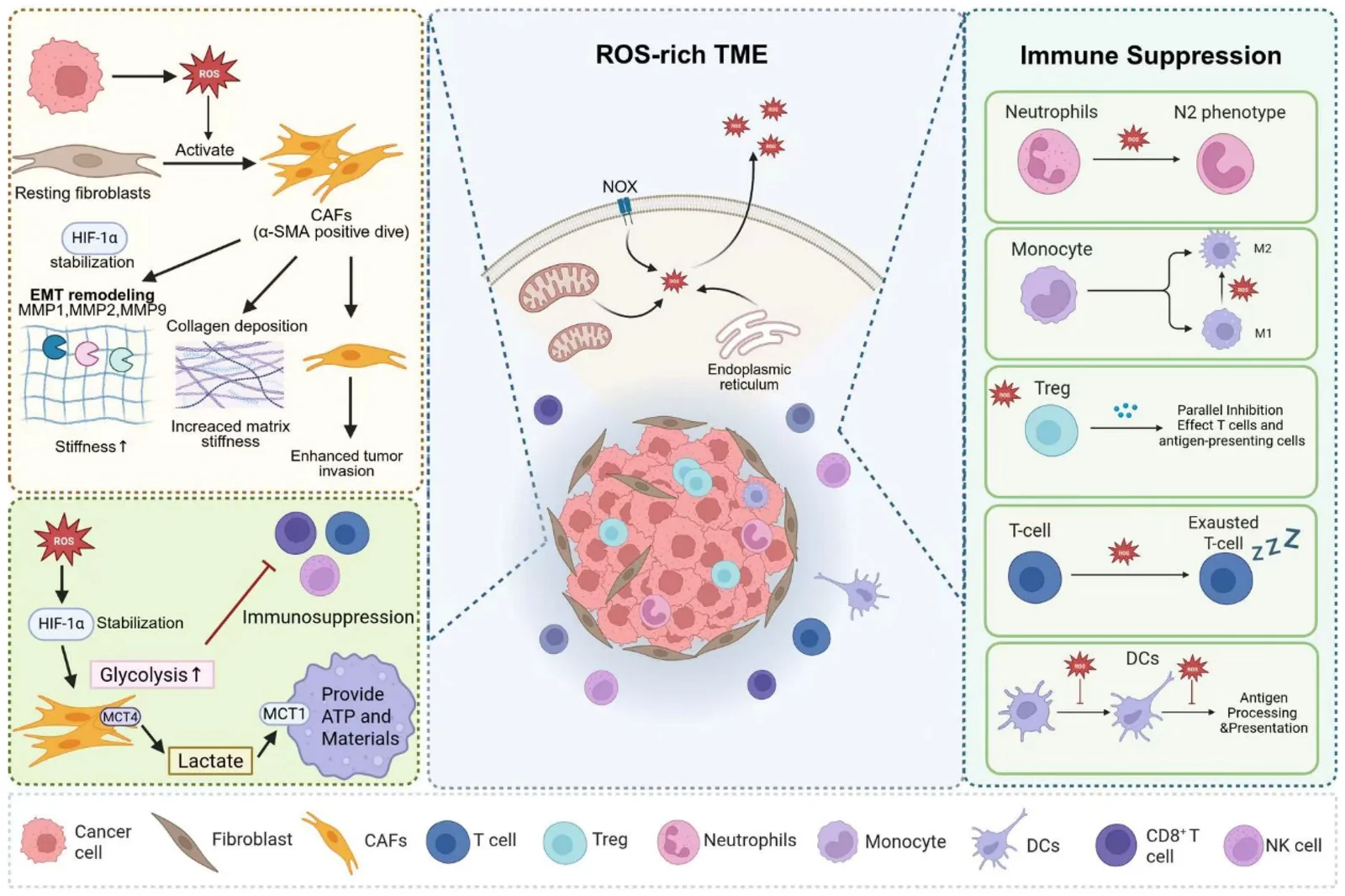
ROS-orchestrated intercellular crosstalk and metabolic-immune remodeling within the TME. Tumor-derived ROS activate CAFs, promote HIF-1α stabilization, enhance ECM remodeling, and drive glycolytic lactate production. Concurrently, elevated ROS polarize tumor-associated macrophages (TAMs) toward an M2-like phenotype and impair T cell and NK cell effector functions, collectively establishing an immunosuppressive and pro-tumorigenic microenvironment.

Once established, CAFs become major amplifiers of oxidative signaling. ROS stabilize HIF-1α in CAFs, leading to increased expression of matrix metalloproteinases MMP-1, MMP-3, MMP-9, and fibrillar collagens [110]. This dual response simultaneously degrades existing ECM barriers and increases matrix stiffness, thereby facilitating tumor cell invasion and collective migration. ROS additionally activate Rho-dependent cytoskeletal remodeling, enhancing CAF contractility and generating physical tracks that support invasive tumor spread (Figure 6) [111].

ROS also retool CAF metabolism, and they push glycolysis while curbing mitochondrial respiration, boosting glucose uptake, lactate production, and MCT4-mediated lactate export [112]. Tumor cells, in turn, take up lactate via MCT1 and feed it into the TCA cycle for ATP generation and lipid synthesis. This coupling sets up a lactate shuttle between CAFs and tumor cells, and also depletes local glucose, which in turn dampens CD8^+^ T cell activation and IFN-γ production (Figure 6).

Beyond fueling tumor metabolism, CAF-derived ROS suppress immunity through a separate route, and they activate STAT3 signaling in monocytes, driving IL-10 and TGF-β production and pushing differentiation toward M2-like tumor-associated macrophages (TAMs) (Figure 6).

#### 4.1.2. Oxidative Stress-Mediated Immune Remodeling Within the TME

ROS reshape both myeloid and lymphoid compartments in the TME. In myeloid cells, elevated ROS tilt neutrophils toward an N2-like immunosuppressive state, marked by higher ARG1 and MMP9, less T-cell support, and more invasive activity [113]. ROS also push macrophages toward M2-like TAMs via STAT3 and PPARγ, boosting IL-10 and TGF-β while curbing TNF-α and IL-12 [114].

Dendritic cells do not escape this influence. Tumor-derived ROS activate STAT3 in DC precursors and set up a ROS-IL-6-STAT3 loop that blocks their maturation into antigen-presenting DCs (Figure 6) [115]. High ROS also impair antigen processing and presentation, nudging DCs toward a tolerogenic state that weakens CD8^+^ T cell priming [62].

Lymphoid cells are also affected. Excess H_2_O_2_ can limit T-cell activation, promote exhaustion-associated features, and suppress IFN-γ and TNF-α production [116]. Oxidative stress further interferes with calcium signaling, attenuating Orai1-mediated calcium entry and downregulating STIM/Orai expression, events that in some settings may compromise NFAT activation and T-cell expansion [117].

NK cells are notably sensitive to oxidative challenge. CAF-derived ROS have been reported to reduce the expression of activating receptors such as NKG2D and NKp30, and to diminish perforin and granzyme production, collectively weakening cytotoxic capacity [118]. Together, these alterations may contribute to a redox-driven immunosuppressive milieu that resists immune attack and conventional therapies.

### 4.2. Metabolic Reprogramming and Redox Adaptation in the TME

The metabolic landscape of the TME is highly heterogeneous. Differences in vascular perfusion, oxygen availability, and nutrient supply, together with extracellular acidification and waste accumulation, create a dynamic and metabolically stressful microenvironment. This environment drives extensive metabolic adaptation in both cancer and stromal cells. Within this adaptive framework, ROS function as important mediators linking hypoxia, nutrient sensing, antioxidant defense, and immune regulation.

#### 4.2.1. Hypoxia–ROS–HIF-1α Feed-Forward Loop

**Figure 7:**
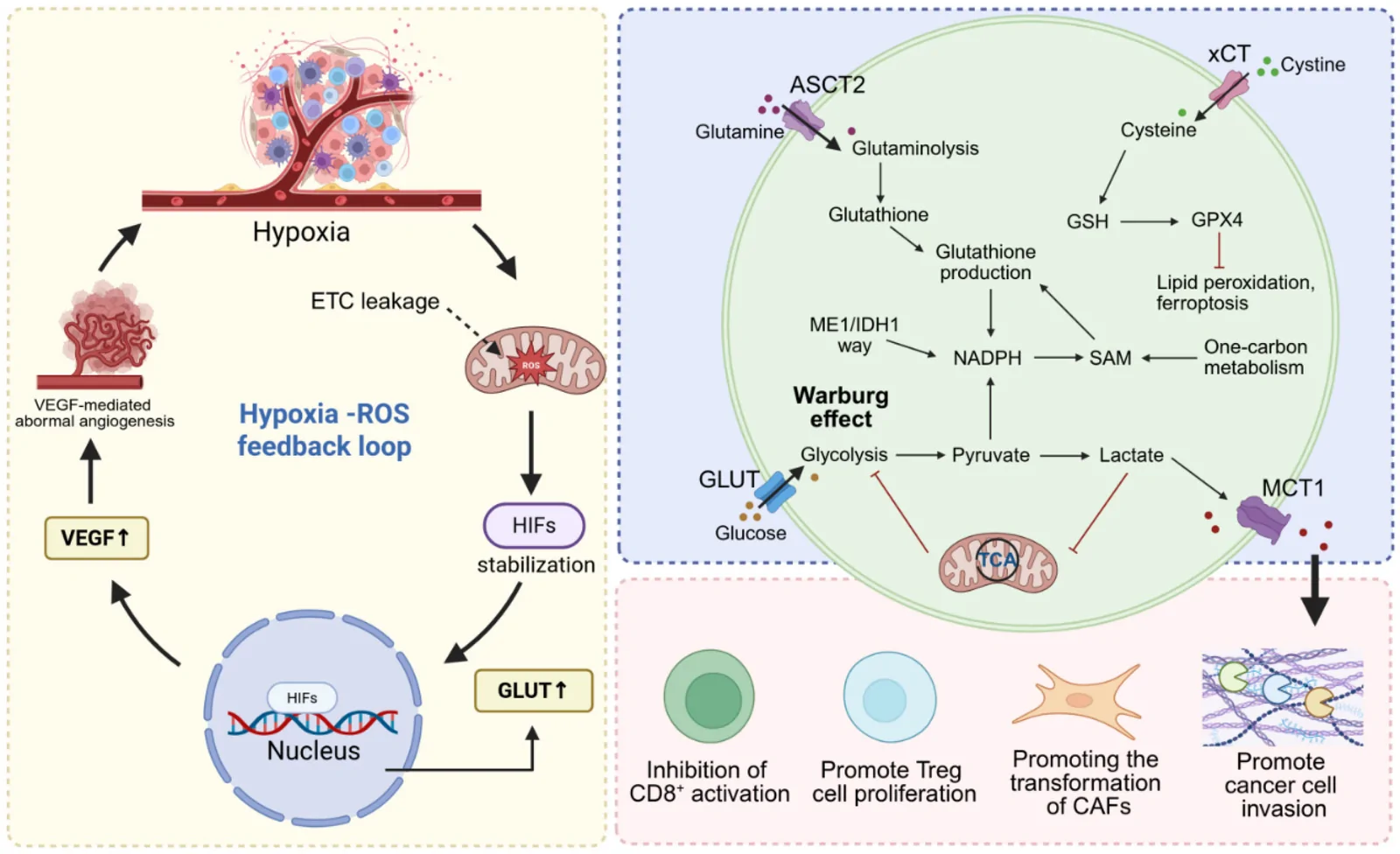
The hypoxia–ROS positive feedback loop and the TME metabolic-redox remodelling network. A self-reinforcing hypoxia–ROS circuit and a coordinated metabolic-redox network reshape the TME. On the one hand, pathological angiogenesis and the mismatch between oxygen supply and demand intensify intratumoral hypoxia. Under these conditions, dysfunction of the mitochondrial electron transport chain promotes electron leakage and excessive ROS generation. Accumulated ROS in the cytosol oxidatively inhibit prolyl hydroxylases (PHDs), preventing HIF-1α hydroxylation and degradation, thereby facilitating its stabilization and nuclear translocation. Nuclear HIF-1α subsequently drives the transcription of genes such as GLUT1 and VEGF. Elevated VEGF further stimulates aberrant angiogenesis, which fails to restore oxygenation and instead exacerbates oxidative stress, reinforcing HIF-1α signaling and establishing a vicious “hypoxia–ROS–HIF-1α–angiogenesis” feedback loop. On the other hand, metabolic reprogramming within the TME supports redox homeostasis and immune evasion. Glutamine uptake via SLC1A5 and serine-driven one-carbon metabolism provide reducing equivalents (NADPH and GSH) to buffer oxidative stress. Meanwhile, Cystine enters cells via SLC7A11 (xCT) and is converted to cysteine, which sustains the GSH-GPX4 axis and keeps lipid peroxidation in check, thereby limiting ferroptosis. Tryptophan metabolism, for its part, generates kynurenine, which activates the aryl hydrocarbon receptor (AHR), dampens effector T cell function, and helps Tregs manage intracellular ROS and survive oxidative stress. These interconnected pathways converge to create a redox-adapted, immunosuppressive environment that favors tumor progression.

Tumor hypoxia arises in part because cancer cells consume oxygen at a rate that exceeds delivery through structurally abnormal and poorly functional vessels [119]. Intermittent perfusion can generate cycles of hypoxia and reoxygenation, thereby intensifying oxidative stress in both tumor and immune compartments. Hypoxia and ROS engage in reciprocal regulation. Under moderate hypoxic conditions, HIF-1α may limit mitochondrial ROS production by suppressing oxidative phosphorylation and enhancing antioxidant programs [120]. In contrast, severe hypoxia or reoxygenation may promote electron leakage from the ETC and a surge in mitochondrial ROS [121]. Elevated ROS have been associated with impaired signaling downstream of the T-cell receptor, reduced NK-cell function, and enhanced immune exhaustion. ROS can also contribute to HIF-1α stabilization by inhibiting prolyl hydroxylases, thereby promoting the expression of glycolytic enzymes, amino acid transporters, and VEGF [122]. The resulting vasculature is often structurally defective and functionally insufficient, which may perpetuate hypoxic conditions while also favoring immunosuppressive myeloid differentiation and Treg activity. Thus, a vicious cycle emerges, namely hypoxia → ROS → HIF-1α stabilization → angiogenesis and immunosuppression → persistent hypoxia (Figure 7).

#### 4.2.2. Lactate as a Metabolic Regulator of Redox Homeostasis and Immune Suppression

Lactate accumulation is a hallmark of aerobic glycolysis in tumors. The enzymatic conversion of pyruvate to lactate by lactate dehydrogenase A (LDHA), followed by its export through monocarboxylate transporter 4 (MCT4), enables tumor cells to reduce mitochondrial oxidative phosphorylation and thereby limit ROS generation (Figure 7) [123]. From this perspective, lactate is increasingly recognized as an active regulator of redox homeostasis rather than a passive metabolic waste product. Lactate can engage the Nrf2–GSH antioxidant axis in both tumor and immune cells, increasing GSH levels and antioxidant enzyme activity and thereby supporting ROS detoxification, as demonstrated in pancreatic adenocarcinoma [124]. This metabolic adaptation confers survival advantages and contributes to therapeutic resistance in tumors, yet its repercussions for immune cells are strikingly divergent (Figure 7). In CD8^+^ T lymphocytes, excess lactate attenuates the physiological ROS signals prerequisite for effective activation, represses Lck phosphorylation, and fosters an exhausted state [125]. Fischer and his co-workers demonstrated that tumor-derived lactate markedly depletes intracellular ROS in T cells, obstructing Lck phosphorylation and diminishing IFN-γ output; notably, replenishment of low-dose ROS partially reinstated T-cell effector functions [125]. Extending these observations, Angelin and colleagues reported that under high-lactate conditions, tumor-infiltrating CD8^+^ T cells substantially upregulate GPX4, which further lowers ROS and drives an exhaustion phenotype defined by elevated PD-1. Pharmacological inhibition of glutathione synthesis, using BSO, for instance, reverses lactate-mediated ROS clearance, restores cytotoxic capacity, and augments antitumor efficacy [126]. Tregs exploit lactate via MCT1 to sustain their proliferative and immunosuppressive functions [127]. Macrophages adopt yet another strategy: lactate signaling through GPR132 suppresses NOX2-derived ROS and promotes M2-like polarization, accompanied by increased ARG1 and interleukin 6 (IL-6) production and reduced inflammatory responses [128]. Additionally, lactate contributes to CAF activation and the construction of an immunosuppressive stromal architecture [129]. Moreover, in pancreatic cancer cells, lactate can be recycled by MCT1-high tumor cells as an alternative carbon source for the TCA cycle, thereby supporting growth, invasion, and metastasis [130].

In summary, lactate accumulation reshapes the TME through two interconnected dimensions—metabolic reprogramming and immune suppression—ultimately promoting tumor progression and metastasis. Accordingly, targeting lactate metabolism may represent a viable therapeutic strategy. Preclinical studies have shown that targeting lactate metabolism can restore T-cell function, reduce CAF activity, and inhibit tumor growth [131], laying the foundation for future development of lactate-targeted therapies.

#### 4.2.3. Amino Acid Metabolism Integrates Redox Homeostasis and Immune Evasion

Glutamine metabolism intersects with redox regulation at multiple levels. Glutamine fuels the tricarboxylic acid (TCA) cycle and, through glutaminolysis and associated metabolic shuttles, contributes to the generation of NADPH and GSH, thereby supporting cellular defense against oxidative stress (Figure 7). Pathways such as the pyruvate–malate shuttle, one-carbon metabolism, and NADPH-generating reactions mediated by enzymes such as malic enzyme 1 (ME1) and isocitrate dehydrogenase 1 (IDH1) can enhance the reducing capacity of tumor cells and facilitate ROS detoxification. Elevated GLS1 expression has been associated with poor prognosis, whereas glutaminase (GLS) inhibition can disrupt both redox homeostasis and ATP production [132]. In a nutrient-deprived xenograft model, CAFs were found to supply glutamine to tumor cells through autophagy, while ambient glutamine simultaneously sustains autophagic flux within the CAFs themselves [133]. Cystine uptake via xCT (SLC7A11) drives GSH synthesis and supports GPX4, forming a key barrier against lipid peroxidation and ferroptosis. High SLC7A11 expression has been linked to unfavorable outcomes and immune evasion [134]. Serine metabolism contributes one-carbon units, NADPH, and nucleotide precursors—pathways especially important for antioxidant defense and biosynthesis under hypoxic conditions.

Tryptophan catabolism through IDO1/TDO yields kynurenine, which engages the aryl hydrocarbon receptor (AHR) to restrain CD8^+^ T-cell proliferation, upregulate PD-1 and TIM-3, and bolster the oxidative stress resistance of Tregs [135]. Guo et al. extended this picture, demonstrating that tumor cells outcompete effector lymphocytes for glutamine, further compromising T-cell function [136].

These metabolic pathways are interconnected and collectively contribute to the establishment of an immunosuppressive redox environment. ROS signaling, hypoxia, lactate metabolism, amino acid availability, and antioxidant systems converge to support tumor growth while limiting antitumor immunity. Collectively, these findings suggest that combined metabolic and redox targeting may represent a promising direction for future therapeutic development.

## 5. Oxidative Stress-Induced Outcomes in Cancer Cells

The preceding sections have emphasized that ROS can exert opposing effects depending on their abundance. At relatively low to moderate levels, ROS activate NF-κB, MAPK, and PI3K/AKT signaling, thereby supporting proliferation, survival, and metabolic adaptation. Within the TME, ROS also modulate stromal and immune cell behavior, often shifting the microenvironment toward immune evasion and impaired antitumor responses. ROS-induced autophagy represents another adaptive mechanism that enables cancer cells to remove damaged organelles, recycle nutrients, and maintain metabolic activity under hypoxic or nutrient-deprived conditions. Collectively, these mechanisms enhance cellular adaptation to stress and contribute to therapeutic resistance.

At higher ROS levels, however, the adaptive responses described above can become detrimental once antioxidant buffering capacity is exceeded. Excessive ROS induce oxidative damage and activate regulated cell death pathways, including apoptosis, necroptosis, and ferroptosis, as well as more recently characterized modalities such as pyroptosis and copper-induced cell death (cuproptosis). The following sections examine the molecular mechanisms underlying these oxidative stress-induced cell death pathways and discuss their potential as therapeutic targets.

### 5.1. Cell Death Patterns

Oxidative stress damages proteins, lipids, and DNA while simultaneously activating multiple regulated cell death pathways. In cancer cells, these pathways are not necessarily discrete but frequently intersect, highlighting the complex relationship between cellular redox status and cell fate.

Pyroptosis provides one example: ROS can activate inflammasomes and gasdermin-mediated pore formation, ultimately leading to the release of inflammatory mediators. Ferroptosis follows a distinct mechanism in which ROS-driven lipid peroxidation, particularly under conditions of iron accumulation, overwhelms GPX4-dependent lipid peroxide detoxification and drives cells toward a non-apoptotic form of cell death. These examples illustrate that the same oxidative stress may engage distinct cell death pathways depending on the underlying molecular context.

Copper ions have recently emerged as additional regulators of oxidative cell death. Copper can bind to components of the TCA cycle, disrupt protein homeostasis, induce proteotoxic stress, and ultimately trigger copper-dependent cell death, or cuproptosis [137]. Apoptosis and necroptosis are likewise influenced by ROS, either through the promotion of mitochondrial dysfunction and caspase activation or, under conditions where caspase activity is limited, through engagement of the RIPK1-RIPK3-MLKL axis that drives necroptotic death. Collectively, these ROS-responsive pathways highlight the multifaceted role of oxidative stress in tumor cell fate regulation and present a range of potential entry points for redox-targeted therapeutic strategies.

#### 5.1.1. Oxidative Stress-Induced Apoptosis

**Figure 8:**
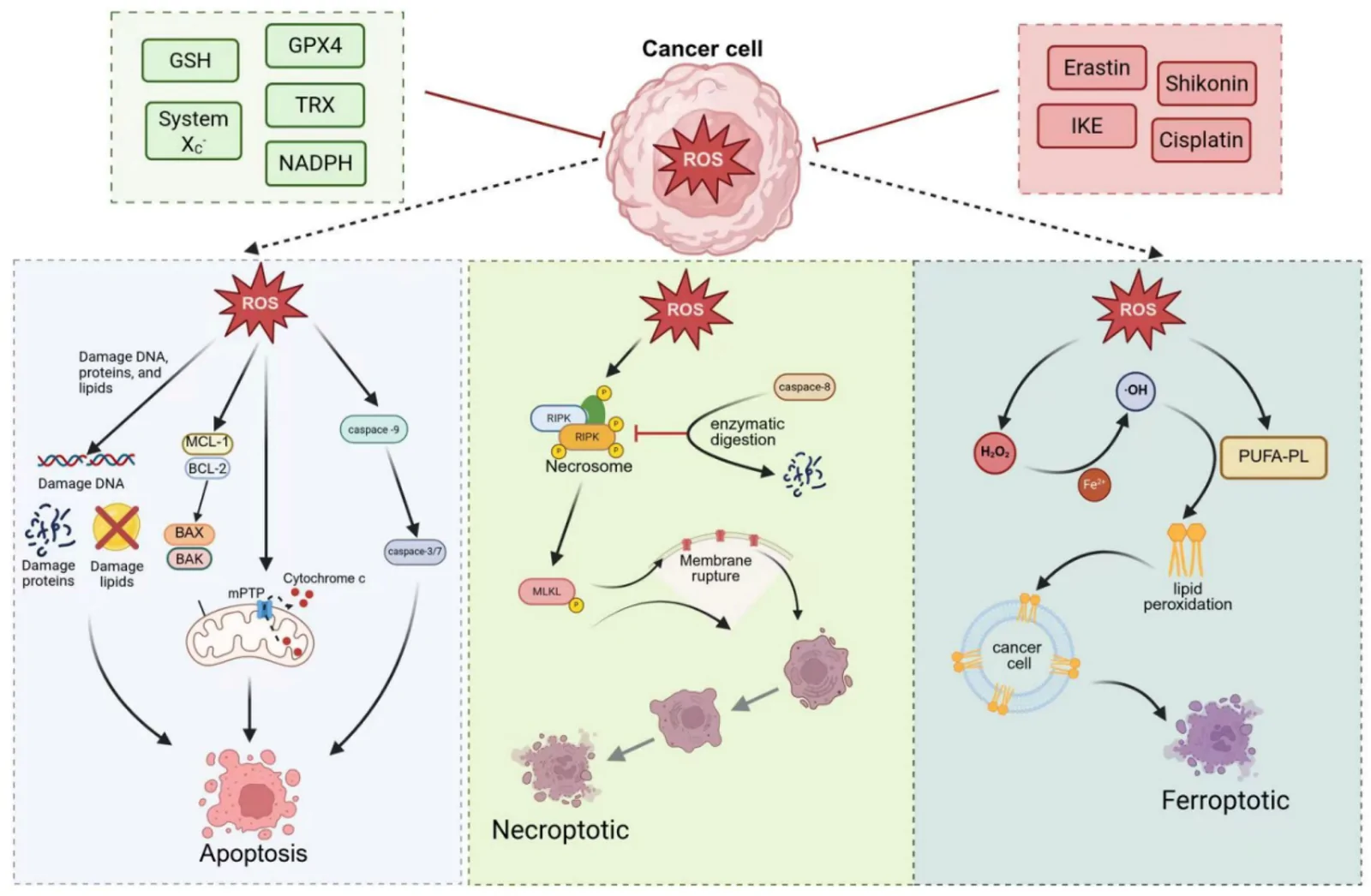
The ROS-Mediated Trifecta: Divergent Mechanisms of Regulated Cell Death. Apoptosis (**Left**): Accumulation of ROS within mitochondria initiates oligomerization of the pro-apoptotic proteins Bax and Bak, an event that compromises the integrity of the outer mitochondrial membrane. Cytochrome c then escapes into the cytosol and engages the Caspase-9/3 cascade, executing a classical apoptotic program that does not provoke an inflammatory response. Necroptosis (**Middle**): When oxidative stress is extreme or apoptosis is blocked, RIPK1 and RIPK3 assemble into the necrosome complex. Phosphorylation of MLKL follows, enabling its translocation to the plasma membrane, where it compromises membrane integrity. The resultant cell lysis releases DAMPs, alerting the surrounding tissue to cellular injury. Ferroptosis (**Right**): An expanded labile iron pool drives Fenton chemistry, generating extensive lipid ROS. When the SLC7A11-GSH-GPX4 antioxidant axis fails to keep peroxidation in check, PUFA-PLs undergo uncontrolled oxidation, disrupting membrane integrity and initiating a non-apoptotic, iron-dependent death pathway.

Apoptosis is a canonical form of regulated cell death (RCD) that is essential for tissue homeostasis and the elimination of damaged cells. ROS function as signaling molecules at low levels but can become cytotoxic when present at high levels. Excessive ROS can induce oxidative modifications of redox-sensitive residues in BCL-2 family proteins, including anti-apoptotic members such as BCL-2 and MCL-1, thereby favoring Bax/Bak activation and mitochondrial outer membrane permeabilization (MOMP) (Figure 8). MOMP facilitates the release of cytochrome c and SMAC, leading to activation of the caspase cascade, including caspase-9, caspase-3, and caspase-7, and ultimately executing apoptosis [138].

Mitochondria function as both sources and amplifiers of ROS during apoptosis. TNF-α binding to TNFR1 can promote mitochondrial ROS production through Romo1, which contributes to caspase-8 and caspase-3 activation and can interact with the regulation of anti-apoptotic proteins such as Bcl-xL and cFLIP, thereby establishing a positive feedback relationship with JNK signaling. Excessive ROS can also promote opening of the mitochondrial permeability transition pore (mPTP), further facilitating cytochrome c release and caspase activation. ROS can additionally act as signaling mediators during apoptosis. For example, PUMA promotes mitochondrial ROS generation through BAX-dependent mechanisms, leading to activation of DNA damage responses, including γ-H2AX, ATM/ATR, and Chk1/Chk2 signaling, as well as JNK activation. These events contribute to the suppression of anti-apoptotic proteins such as Bcl-xL and Mcl-1 and ultimately promote apoptosis [139]. Tumor cells often evade ROS-mediated apoptosis by upregulating antioxidant systems (GSH, Trx) or anti-apoptotic proteins (BCL-2, MCL-1), contributing to chemoresistance [140].

#### 5.1.2. Oxidative Stress-Induced Non-Apoptotic Programmed Cell Death

Non-apoptotic ROS-mediated cell death includes necroptosis, ferroptosis, pyroptosis, and copper-induced death. Unlike apoptosis, these pathways are less dependent on caspases and often involve membrane rupture, organelle swelling, and release of DAMPs, generating inflammatory microenvironments. As is currently known, chemotherapeutic agents, radiotherapy, and certain metal ions (e.g., Cu^2+^, Fe^2+^) can exacerbate ROS production by promoting the Fenton reaction, thereby inducing non-programmed necrosis in tumor cells [141,142].

Necroptosis: Inhibition of caspase-8 or loss of cFLIP can facilitate RIPK1 activation, RIPK3 phosphorylation, and MLKL oligomerization, leading to necrosome formation and subsequent necroptotic cell death. ROS can both promote and amplify necroptotic signaling by enhancing RIPK1/RIPK3 activation and lipid peroxidation. Mitochondria-associated membranes (MAMs) may contribute to ROS generation through mitochondrial complex II, thereby sustaining RIPK1/RIPK3 signaling, promoting ATP depletion, and ultimately facilitating plasma membrane rupture (Figure 8).

Ferroptosis: Iron-dependent lipid peroxidation driven by ROS leads to peroxidation of PUFA-containing phospholipids. GPX4 inhibition or depletion impairs detoxification, while Fenton chemistry amplifies ·OH formation. Lipid peroxides decompose into reactive aldehydes (4-HNE, MDA), damaging macromolecules and membranes. Ferroptosis inducers (e.g., RSL3, erastin, sulfasalazine) sensitize tumor cells, including apoptosis-resistant populations, to chemotherapy, radiotherapy, and immunotherapy (Figure 8).

### 5.2. The Resistance of Cancer Cells to Death

Tumor cells undergo adaptive redox reprogramming to survive persistent oxidative stress by integrating enzymatic antioxidant systems, including SOD, catalase, and GSH, with ferroptosis-resistance mechanisms involving system Xc^−^/GPX4 and FSP1, as well as lipid remodeling pathways regulated by ACSL4 and LPCAT3. Autophagy further promotes cell survival by removing damaged organelles and maintaining metabolic homeostasis. Beyond individual tumor cells, this ROS-driven adaptive response interacts with stromal components to establish a microenvironment that supports tumor cell survival and metabolic adaptation. Collectively, these interconnected mechanisms contribute to chemoresistance and resistance to oxidative stress-induced cell death and highlight the functional interplay between autophagy and redox regulation.

#### 5.2.1. Adaptive Upregulation of Antioxidant Systems

To counteract ROS-driven apoptosis, necroptosis, and ferroptosis, cancer cells enhance the activity of antioxidant systems, including SOD, catalase (CAT), and the GSH system (Figure 8). These antioxidant systems provide reducing capacity to detoxify ROS and lipid peroxides, thereby promoting tumor cell survival. Peng and colleagues showed that NF-κB reinforces this antioxidant response by upregulating both the catalytic (GCLC) and modifier (GCLM) subunits of γ-glutamylcysteine ligase, thereby enhancing GSH synthesis [143]. System Xc^−^ (SLC7A11/SLC3A2) imports cystine to fuel GSH synthesis, while FSP1 provides a GPX4-independent backup for ferroptosis suppression. Lipid metabolism also shapes ferroptosis susceptibility, ACSL4 and LPCAT3 govern PUFA incorporation into membranes, and LOX-mediated peroxidation contributes to regulated cell death, though it is not the sole determinant. Deletion or inhibition of ACSL4 lowers PUFA content enough to allow GPX4-null cells to survive long-term [144]. Interestingly, the effects of LOX inhibitors may owe more to their radical-trapping activity than to LOX blockade per se, pointing to non-enzymatic autooxidation as a central driver of ferroptosis execution [145]. In response to diverse ROS-induced death pressures, tumor cells integrate antioxidant pathways with lipid-regulating enzymes to establish a flexible defense network. This redox plasticity not only helps cancer cells evade apoptosis and ferroptosis but also provides a metabolic and signaling basis for resistance to radiotherapy, chemotherapy, and targeted therapies.

#### 5.2.2. Synergistic or Antagonistic Effects of Autophagy and Oxidative Stress

The crosstalk between autophagy and ROS is highly dependent on the extent and duration of oxidative stress. At moderate ROS levels, ROS-induced autophagy generally exerts a protective function by removing damaged organelles and maintaining redox homeostasis. When oxidative stress becomes excessive or sustained, however, autophagic responses may produce divergent outcomes, promoting either cell survival or cell death depending on the cellular context. ROS can induce autophagy through multiple upstream mechanisms, including AMPK activation, mTOR inhibition, JNK signaling, and the p62/Keap1/Nrf2 axis [146]. In turn, autophagy limits ROS accumulation by removing damaged mitochondria (mitophagy), clearing oxidized proteins and aggregates, and supporting metabolic pathways that regenerate NADPH and GSH [146].

In genetically engineered mouse models, Taguchi et al. found that mitophagy selectively removes ROS-generating mitochondria, while autophagic degradation of p62 regulates Nrf2 signaling [147]. Specifically, autophagy contributes to redox homeostasis through continuous turnover of p62. When autophagy is impaired, for instance by ATG7 deletion, p62 accumulates in the cytoplasm and subsequently enters the nucleus, where it triggers Nrf2 activation [148]. In established tumors, autophagy can therefore support redox adaptation by facilitating the removal of damaged cellular components and helping tumor cells tolerate oxidative stress. Autophagy also contributes to metabolic adaptation, epithelial–mesenchymal transition (EMT), and metastasis. Sun et al. reported that, in metastatic melanoma, SIRT1 promoted autophagic degradation of E-cadherin by deacetylating Beclin 1, thereby inducing EMT and enhancing cell migration and invasion. Conversely, autophagy inhibition restored E-cadherin expression and suppressed migration and invasion in cells overexpressing SIRT1 [149]. Not only that, but autophagy can also use PDGFR-β/Cav-1 to enhance glycolysis in CAFs via autophagy-mediated metabolic reprogramming, leading to increased lactate output and then promoting the development of breast carcinoma cells [150]. Disruption of autophagy, particularly through knockout of the key autophagy gene ATG7, leads to the accumulation of dysfunctional mitochondria and elevated ROS levels, thereby causing the death of cancer cells [151]. Notably, autophagy can also actively promote cell death under specific contexts. For example, ferritinophagy, a selective form of autophagy, degrades ferritin and increases the labile iron pool, thereby sensitizing cells to ferroptosis [152]. This dual capacity—protective under moderate oxidative stress but potentially deleterious under excessive or sustained stress—underscores the context-dependent nature of the autophagy–ROS axis.

**Figure 9:**
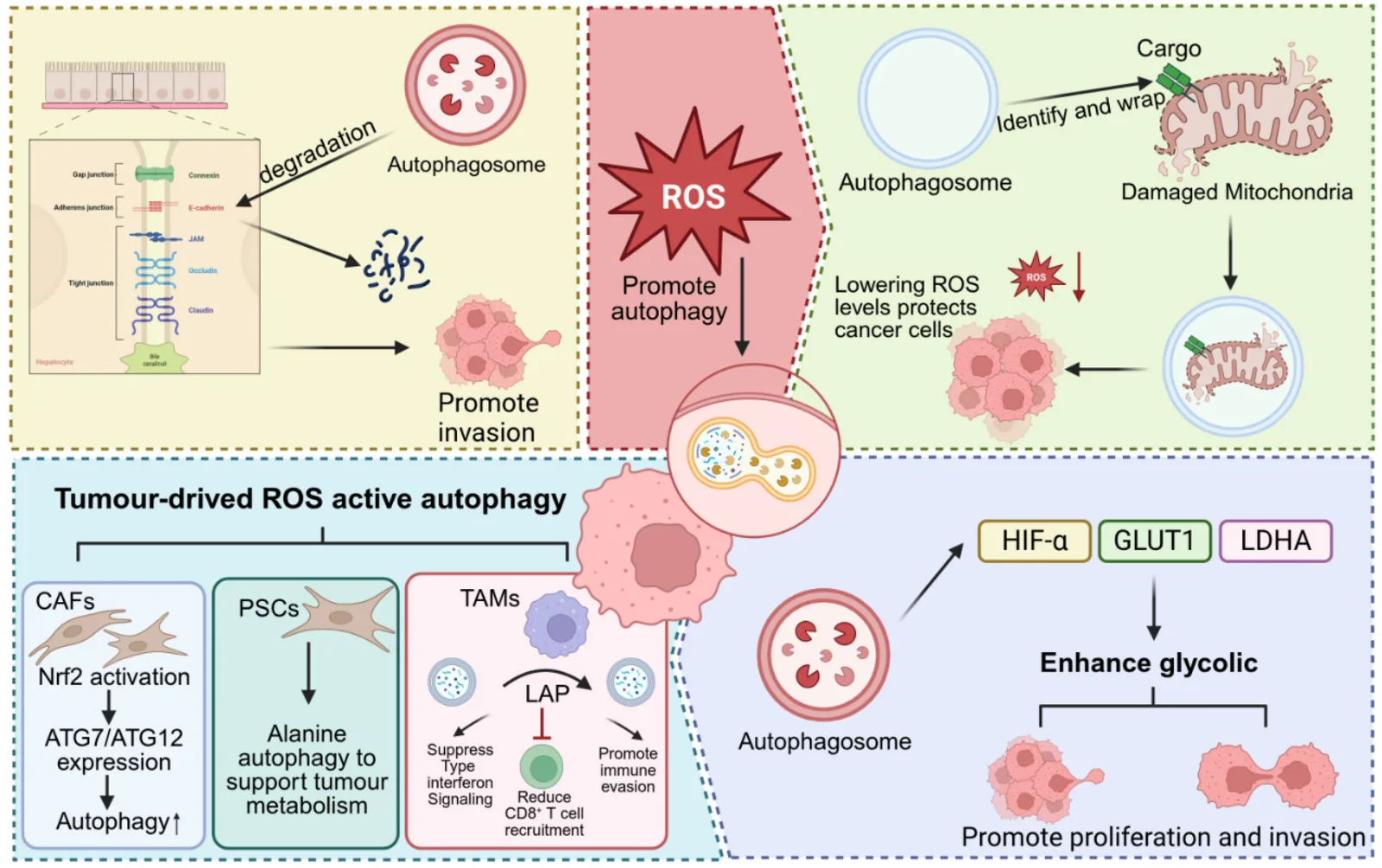
Context-dependent interplay between autophagy and ROS in tumor progression and the TME. ROS and autophagy form a dynamic regulatory axis with stage- and context-specific functions in cancer. Moderate ROS levels act as signaling cues to induce autophagy, facilitating the clearance of damaged organelles, particularly mitochondria, via mitophagy, thereby restraining intracellular ROS accumulation and preserving redox homeostasis. In contrast, sustained oxidative stress or impaired autophagic flux disrupts this balance, leading either to excessive degradation or defective turnover, both of which can contribute to cell death or tumor-promoting signaling. In tumor cells, autophagy supports progression by maintaining mitochondrial quality control and metabolic adaptation. Selective autophagic degradation of adhesion molecules such as E-cadherin weakens intercellular junctions and promotes invasive behavior. Concurrently, autophagy sustains a tolerable ROS threshold that enables activation of redox-sensitive pathways (e.g., HIF-α-GLUT1-LDHA), thereby enhancing glycolytic reprogramming, proliferation, and invasion. ROS also reshapes the tumor microenvironment (TME) by inducing autophagy in stromal components. In TAMs, LC3-associated phagocytosis (LAP) sustains ROS-dependent signaling, curbs type I interferon responses, and dampens CD8^+^ T cell recruitment, collectively reinforcing immune evasion. CAFs and PSCs take a different route: ROS-driven Nrf2 activation there upregulates ATG7 and ATG12, promoting the release of alanine and other metabolites that feed into tumor metabolic demands.

ROS also modulate autophagy in stromal cells within the TME, including CAFs, pancreatic stellate cells (PSCs), and tumor-associated macrophages (TAMs). In these cells, ROS can activate autophagy through Nrf2-mediated antioxidant signaling and LC3-associated phagocytosis (LAP), thereby promoting the turnover of damaged cellular components and the secretion of metabolites such as alanine [153], which can be utilized by adjacent tumor cells to support anabolic metabolism and energy production. Concurrently, ROS-induced autophagy in stromal immune cells suppresses CD8^+^ T cell recruitment and effector function, contributing to immune evasion and creating a metabolically and immunologically permissive niche that favors tumor survival [154] (Figure 9). Therapeutically, strategies that combine autophagy inhibitors, targeting key regulators such as ULK1/2 and ATG7, with ROS-inducing agents have shown preclinical efficacy in sensitizing tumors to chemotherapy and immunotherapy by disrupting this protective stromal support [155].

## 6. Advances in Anticancer Drugs Targeting Oxidative Stress

Oxidative stress has emerged as an important therapeutic target in cancer because of its close interplay with metabolic reprogramming, immune regulation, and treatment response. Redox-based therapeutic strategies have evolved beyond the conventional dichotomy between antioxidant and pro-oxidant approaches, increasingly focusing on tumor-selective and context-dependent modulation of redox homeostasis. One strategy is to increase ROS levels beyond the tolerance threshold of cancer cells, whereas another is to disrupt the antioxidant systems that enable tumor cells to withstand oxidative stress. In parallel, modulation of ROS signaling within the TME may enhance antitumor immunity and improve the efficacy of conventional anticancer therapies. Both natural and synthetic compounds have been investigated in this context, with accumulating evidence supporting their potential as redox-targeted anticancer strategies.

### 6.1. Natural Medicines

Natural products represent a diverse source of redox-active compounds with potential anticancer activity. Their structural diversity enables them to modulate redox homeostasis through coordinated effects on mitochondrial function, redox-regulatory pathways, and immune checkpoint signaling. Such multitarget activities may be difficult to achieve with conventional single-target agents.

#### 6.1.1. Polyphenolic Compounds

Plant-derived polyphenols constitute a structurally diverse class of compounds characterized by the presence of multiple phenolic hydroxyl groups. Traditionally, these compounds have been regarded primarily as antioxidants. However, accumulating evidence indicates that they can act as dynamic redox modulators, exhibiting antioxidant or pro-oxidant effects depending on the cellular context. Because basal ROS levels are already elevated in many tumor cells, polyphenols may further increase oxidative stress beyond the threshold tolerated by cancer cells, thereby inducing cell death.

Structurally diverse polyphenolic and related phytochemicals include flavonoids such as quercetin and kaempferol, stilbenes such as resveratrol, catechins such as epigallocatechin gallate (EGCG), and anthraquinones such as emodin. Despite differences in their chemical structures, these compounds share several common mechanisms of action, including modulation of mitochondrial function, regulation of glutathione (GSH) metabolism and turnover, and activation of ROS-sensitive signaling pathways. Curcumin and EGCG are among the most extensively studied compounds in this group and have advanced to clinical or preclinical development (Table 1) [156]. Curcumin exhibits a concentration-dependent ROS profile; under normal or mildly oxidative conditions, it activates Nrf2 and upregulates HO-1 and NQO1 [156]. In tumor cells, it tends to shift toward ROS-dependent suppression of malignancy, engaging the ROS/KEAP1/Nrf2 axis, inducing miR-34 family members, and restraining colorectal cancer progression [157]. At the same time, curcumin hits cancer stem cells across multiple pathways (Hedgehog, Notch, etc.), curtailing self-renewal and countering therapy resistance [158]. EGCG operates through a related mechanism, modulating oxidative stress via mitochondrial and inflammatory pathways. By attenuating CCL5-driven ROS production and restoring KEAP1-Nrf2 equilibrium, it reduces Cyclin D1 and PCNA expression and restricts tumor growth [159]. Other polyphenols such as resveratrol, quercetin, and sulforaphane exhibit comparable redox-dependent antitumor properties [160,161].

Overall, polyphenols are increasingly viewed as context- and threshold-dependent redox modulators rather than simple ROS scavengers. Their clinical translation remains challenging because of limited bioavailability, rapid metabolic clearance, and redox effects that vary according to the cellular and microenvironmental context.

#### 6.1.2. Terpenoids

Terpenoids are grouped by carbon skeleton into monoterpenes (C10), sesquiterpenes (C15), diterpenes (C20), and triterpenes (C30) [173], and they represent a major category of natural products with redox-modulating properties. A recurring theme among these compounds is their ability to exploit the elevated oxidative state typical of tumor cells, either by amplifying ROS production or by undermining antioxidant defenses.

Artemisinin and its derivative dihydroartemisinin (DHA) serve as instructive examples (Table 1). Their endoperoxide bridge reacts with intracellular Fe^2+^ to generate carbon-centered radicals, which can drive ROS accumulation, oxidative stress, DNA damage, and ER stress, events that may ultimately lead to apoptosis [164]. Under GPX4-deficient conditions, DHA has also been reported to sensitize cells to ferroptosis, an effect linked to elevation of the labile iron pool and increased lipid peroxidation [164]. Thymoquinone (Table 1) operates through a copper-dependent redox cycle, generating ROS at DNA-associated sites and provoking oxidative DNA damage and apoptosis [165]. Other monoterpenoids, such as thymol, disrupt mitochondrial respiration and promote ROS accumulation. Triterpenoids like ursolic acid take a different approach, suppressing antioxidant enzymes and Nrf2 signaling to exacerbate oxidative injury (Table 1) [174]. Triptolide and betulinic acid likewise induce ROS overload and mitochondrial dysfunction (Table 1) [167,168].

Despite their structural diversity, these terpenoids converge on a common functional outcome: destabilizing the redox balance that tumor cells depend on, thereby steering them toward oxidative death. This convergence highlights a broader therapeutic principle: precision redox modulation may target a core vulnerability of cancer cells while largely sparing normal tissues.

#### 6.1.3. Quinones

Quinones are redox-active metabolites characterized by a conjugated cyclic diketone structure and produced by plants, fungi, and microorganisms. This structural feature enables efficient redox cycling and sustained ROS generation. Following one-electron reduction, the resulting semiquinone radical can react with molecular oxygen to generate O_2_·^−^, H_2_O_2_ and OH, thereby creating a sustained oxidative environment. β-Lapachone illustrates how tumor-selective exploitation can be achieved (Table 2). In tumors that overexpress NQO1, futile redox cycling consumes NADPH, generates substantial ROS, induces DNA fragmentation, and overactivates PARP1 [175]. The resulting depletion of NAD^+^ and ATP triggers bioenergetic collapse. β-Lapachone also induces immunogenic cell death, accompanied by release of ATP, HMGB1, and calreticulin, which may potentiate dendritic cell activation as well as CD8^+^ T cell responses [176].

Shikonin and related naphthoquinones compromise mitochondrial respiratory function and can provoke apoptosis or necroptosis (Table 1). In glioblastoma models, ROS-dependent reinforcement of RIP1/RIP3 signaling may drive cells toward caspase-independent necroptosis [170]. Other quinones, including juglone and menadione derivatives, appear to act through similar redox-cycling and mitochondrial disruptive mechanisms [171,177]. Clinical translation of these agents will require improved delivery strategies and more effective targeting of tumor tissues.

### 6.2. Synthetic Drugs and Targeted Oxidative Stress Strategies

Although synthetic drugs offer advantages in target specificity, pharmacokinetic properties, and opportunities for rational optimization, many anticancer agents are derived from natural products or developed as semisynthetic analogs. Natural and synthetic compounds therefore represent complementary sources for the development of redox-targeted therapies. Synthetic strategies can further enable more precise manipulation of specific redox vulnerabilities, including ROS generation, antioxidant defenses, and redox-sensitive signaling pathways.

#### 6.2.1. ROS Inducers

Some synthetic compounds have been developed to overwhelm the oxidative threshold of cancer cells. Elesclomol, a copper-binding compound, delivers Cu^2+^ into mitochondria; once released, it promotes ROS generation, depletes glutathione, and triggers a copper-dependent form of cell death (Table 2) [137]. In glioblastoma models, this agent sensitizes tumor cells to TMZ and suppresses glioma stem cell populations [178].

ME-344 acts through a mechanistically distinct pathway. Currently being evaluated in early-phase trials, this inhibitor targets mitochondrial complex I and impairs bioenergetic function (Table 2). The downstream effects include ROS accumulation, mitochondrial depolarization, impaired mitophagy, and apoptosis [179]. Additional ROS-generating agents rely on varied mechanisms. β-Lapachone exploits futile redox cycling in tumors that overexpress NQO1 [180]. Menadione generates ROS continuously via semiquinone-mediated electron transfer, leading to mitochondrial dysfunction and ATP depletion [177]. AT-101 combines inhibition of anti-apoptotic BCL-2 family members with ROS-mediated mitochondrial damage, and has been shown to sensitize tumors to chemotherapy and radiotherapy (Table 2) [181]. The capacity of these compounds to amplify oxidative stress in a relatively selective manner may underlie their ability to overcome resistance and promote tumor cell elimination.

#### 6.2.2. Antioxidant System Inhibitor

An alternative approach to targeting tumor redox balance is to disrupt the endogenous antioxidant systems on which cancer cells depend. Rather than directly increasing ROS production, these agents impair the capacity of tumor cells to tolerate and detoxify oxidative stress.

APR-246 acts through this mechanism by covalently modifying cysteine residues and depleting intracellular glutathione (Table 2). In therapy-resistant lymphoma models, APR-246 has been shown to synergize with asparaginase, thereby enhancing lipid peroxidation and promoting ferroptosis [182]. Auranofin, originally a rheumatoid arthritis drug, acts by irreversibly inhibiting thioredoxin reductase (TrxR) through covalent targeting of its selenocysteine residue (Table 2) [183]. TrxR blockade results in ROS accumulation and, in some contexts, global redox disruption. Antitumor activity has been noted across several malignancies, including glioblastoma, lung, pancreatic, ovarian, and hematological cancers [184].

An alternative entry point is transcriptional regulation of antioxidant defenses. Nrf2 governs genes involved in GSH synthesis, ROS detoxification, and xenobiotic metabolism. ML385 disrupts Nrf2 DNA binding and thus suppresses its transcriptional output (Table 2) [185]. A more direct approach involves ferroptosis induction. GPX4 is essential for detoxifying lipid peroxides; its irreversible inhibition by RSL3 (Table 2) triggers rapid lipid peroxide accumulation and regulated cell death [172]. Erastin and its analogues block SLC7A11-mediated cystine uptake, leading to GSH depletion and GPX4 inactivation (Table 2) [186].

Additional strategies target NADPH production, thereby limiting the reducing equivalents available to both GSH- and Trx-dependent antioxidant systems. Inhibitors of the pentose phosphate pathway (PPP) or one-carbon metabolism, including 6-aminonicotinamide and SHIN1, have been shown to increase oxidative stress and enhance sensitivity to ferroptosis [187,188]. Together, these findings point to a transition from single-target interventions toward integrated disruption of tumor redox networks.

**Table 2:** Synthetic ROS-targeting drugs.

Compound	Class	Molecular Structures	Clinical Applications	Ref.
β-Lapachone	Naphthoquinone derivative	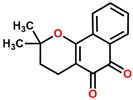	Pancreatic, prostate	[175]
Menadione (Vit K3)	Quinone	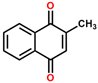	Leukemia, breast	[177]
Elesclomol	Synthetic ROS inducer	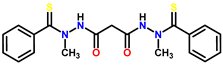	Melanoma, GBM	[178]
ME-344	Isoflavone derivative	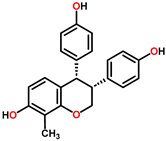	Lung, pancreatic	[179]
AT-101 (R-gossypol)	Small-molecule inhibitor	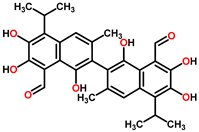	Prostate, lung	[181]
Auranofin	Gold compound	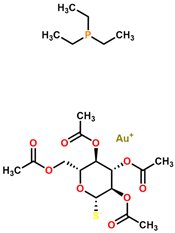	Leukemia, ovarian	[183]
APR-246	p53 reactivator (small molecule)	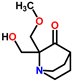	Leukemia (AML, MDS), solid tumors	[182]
ML385	Nrf2 inhibitor	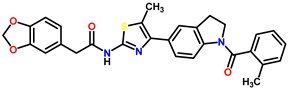	Lung, liver, breast	[185]
Erastin	Ferroptosis inducer	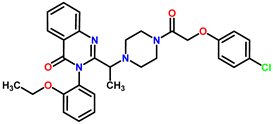	Pancreatic, lung, glioma	[186]
6-AN	NADP^+^ analog/PPP inhibitor	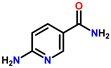	Breast, lung, leukemia	[187]
SHIN1	SHMT1/2 inhibitor	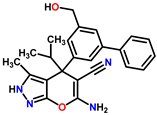	Leukemia, lymphoma	[188]

### 6.3. Microbiome-Mediated Redox Regulation and ROS-Responsive Therapeutic Platforms

The microbiome is increasingly recognized as an important determinant of tumor redox biology. Microbial metabolites can regulate ROS production, antioxidant dependence, ferroptosis sensitivity, and immune function, thereby influencing tumor progression and shaping responses to anticancer therapy.

#### 6.3.1. Effects of Gut Microbiome Metabolites on Tumors

Specific microbial taxa, including Bifidobacterium and Lactobacillus, contribute to systemic redox regulation through vitamin and bioactive metabolite production [189]. This has drawn attention to microbiota-derived metabolites as modulators of oxidative stress.

Gut microbiota-derived metabolites, such as butyrate and secondary bile acids, can modulate vitamin D receptor (VDR) signaling. Activation of VDR suppresses SLC7A11 expression, reduces cysteine uptake, and weakens GPX4-dependent antioxidant capacity, thereby promoting lipid peroxidation and ferroptosis in tumor cells. This provides a mechanistic link between the gut microbiome and tumor redox regulation [190]. Activation of the vitamin D receptor (VDR) has been shown to suppress SLC7A11 expression, reduce cystine uptake, and weaken GPX4-dependent antioxidant capacity in multiple tumor types (Table 3) [190]. The resulting lipid peroxide accumulation may enhance ferroptosis susceptibility, particularly in colorectal cancer stem cells. Butyrate, a short-chain fatty acid produced by Faecalibacterium prausnitzii and Roseburia species, acts as an HDAC inhibitor and modulates GPX4 and SLC7A11 expression (Table 3) [191]. Through promotion of lipid ROS accumulation, butyrate sensitizes tumors to ferroptosis inducers, radiotherapy, and chemotherapy. Depletion of butyrate-producing bacteria reduces ferroptosis susceptibility, while supplementation restores therapeutic responsiveness. Deoxycholic acid exerts context-dependent effects: chronic exposure drives ROS-mediated DNA damage and carcinogenesis, whereas acute exposure may transiently increase oxidative vulnerability in established tumors (Table 3) [192]. These observations underscore the dualistic and highly context-specific nature of microbiome-derived metabolites.

#### 6.3.2. ROS-Responsive Nanocarriers and Combination Therapy Strategies

The persistently oxidative tumor microenvironment has driven the development of ROS-responsive drug delivery systems (DDSs). These carriers typically incorporate ROS-labile chemical linkers or functional groups that undergo structural changes upon ROS exposure, enabling controlled cargo release. Common designs include thioether- or thioketal-based linkers, as well as diselenide bonds, boronic esters, and oxalate derivatives [194]. Oxidative cleavage of these linkers destabilizes the nanocarrier and allows drug release preferentially within tumor tissues. Diselenium-based systems are particularly sensitive because they respond to both ROS and GSH, while boronic esters tend to exhibit greater selectivity toward H_2_O_2_.

Beyond simple delivery, newer platforms have integrated additional functionalities. Co-encapsulation of ferroptosis inducers, GPX4 inhibitors, or iron-based catalysts can establish self-reinforcing oxidative circuits that may lead to irreversible redox catastrophe [195]. Photodynamic therapy has also been combined with these systems, as ROS generated upon light activation can simultaneously trigger drug release and exacerbate oxidative damage (Table 4). Some evidence suggests that ROS-responsive platforms might improve immunotherapy outcomes, possibly through induction of immunogenic cell death and modulation of immune-cell redox states [196].

However, the heterogeneous spatial distribution and temporal dynamics of ROS within tumors may affect therapeutic consistency, while unintended activation in inflamed or oxidatively stressed normal tissues could reduce selectivity. The long-term safety of metal-containing nanomaterials also requires careful evaluation.

Addressing these challenges may require multi-stimuli-responsive designs, selective targeting of specific ROS, and combination with inhibitors of Nrf2, thioredoxin reductase, or ferroptosis-regulatory pathways. Such approaches may improve the precision of redox-based cancer therapy and facilitate its clinical translation.

### 6.4. Combinatorial Targeting of ROS and the Tumor Microenvironment

The efficacy of redox-targeted therapy is strongly influenced by the tumor microenvironment. Stromal and immune cells, including CAFs, TAMs, Tregs, and neutrophils, both produce and respond to ROS, creating a heterogeneous redox landscape that can limit the effectiveness of single-agent ROS modulation. In a study by Geng and his co-workers, they found that the ROS-responsive R848 prodrug (R–P) stays stable in circulation but releases R848 in the oxidative TME. Mannose nanoparticles (R–P@APM-DPs) repolarize TAMs via CD206/TLR7/8, enhancing anti-PD-1 therapy [199]. Moreover, as discussed above, hypoxia, lactate accumulation, and metabolic competition further reshape redox homeostasis, often attenuating ROS-induced cytotoxicity and promoting immune evasion. Therefore, rational combination strategies are required to simultaneously target tumor-intrinsic redox vulnerabilities and TME-mediated resistance.

Several combinatorial approaches have shown preclinical promise. ROS-inducing agents can be combined with immune checkpoint inhibitors to enhance immunogenic cell death and reverse immunosuppression. Ferroptosis inducers, such as GPX4 inhibitors or erastin, sensitize tumors to radiotherapy and chemotherapy [186,200]. Inhibitors of Nrf2, TrxR, or GSH synthesis can overcome adaptive antioxidant responses when combined with targeted therapies [143,184,201]. In addition, ROS-responsive nanocarriers enable tumor-selective drug release and can be integrated with photodynamic therapy or immunotherapy to amplify oxidative damage while reducing systemic toxicity [21]. These strategies highlight the importance of considering the TME as both a barrier and a therapeutic target in redox-based combination regimens. The typical therapies and their association with oxidative stress are shown in Table 4.

## 7. Conclusions and Outlook

ROS are no longer regarded simply as metabolic byproducts. In current understanding, they lie at the intersection of oncogenic signaling, metabolic adaptation, immune regulation, and cell-fate control. Throughout malignant progression, ROS participate in cellular transformation, contribute to TME remodeling, and influence tumor responses to treatment. They are not nonspecific toxins; rather, their effects depend on concentration, subcellular localization, timing, and the state of the target cell.

More important than the total ROS burden may be the ability of tumor cells to maintain redox conditions within a range that permits growth while remaining below the threshold of lethality. Moderate oxidative stress, acting through reversible post-translational modifications, supports proliferation, survival, stem-like properties, and therapeutic resistance. Once this system is overwhelmed, however, the outcome shifts: ROS accumulation can trigger ferroptosis, apoptosis, pyroptosis, or necroptosis. Survival under such oxidative pressure therefore requires an integrated adaptive network encompassing NADPH supply, GSH recycling, Trx function, Nrf2-driven transcription, mitochondrial quality control, and metabolic exchange with the TME. This interconnected network creates a form of redox dependency that resembles non-oncogene addiction and may therefore represent a therapeutically exploitable vulnerability.

The influence of redox regulation extends beyond tumor cells. Oxidative stress can remodel the functions of CAFs, TAMs, neutrophils, endothelial cells, and cytotoxic lymphocytes, with consequences for nutrient availability, immune surveillance, vascular support, and therapeutic sensitivity. Microbiota-derived metabolites and stromal interactions may also shape tumor-specific redox environments. ROS therefore operate at both intracellular and intercellular levels. This duality may help explain why redox-targeted interventions often affect not only cancer cell-intrinsic pathways but also the surrounding microenvironment.

Several redox-oriented therapeutic strategies have emerged from this mechanistic framework. Early-phase and preclinical studies have reported activity across approaches ranging from ROS elevation and antioxidant suppression to ferroptosis induction, mitochondrial disruption, and ROS-responsive delivery systems. The broader shift has been from broad-spectrum redox manipulation toward more selective targeting of tumor-specific dependencies, with oxidative stress increasingly viewed as a system-level vulnerability. Nonetheless, clinical translation remains challenging. Redox states vary considerably across tumor sites, time points, and individual patients, and validated biomarkers are still lacking. Tumors can also activate compensatory antioxidant pathways when one redox pathway is inhibited, limiting the efficacy of single-agent therapies. In addition, many pro-oxidant compounds have narrow therapeutic windows, raising concerns about damage to normal tissues. Moving forward, a clearer understanding of how redox networks are organized and how they adapt during tumor progression and treatment will be required.

Systems-level approaches and precision medicine will likely shape the next phase of this field. Reliable biomarkers, real-time monitoring, and computational modeling of metabolic and antioxidant fluxes could help stratify patients and guide treatment design. Rational combination strategies, including immunotherapy combined with ferroptosis activators, DDR inhibitors, or ROS-responsive nanocarriers, may help overcome resistance and improve treatment tolerability. The tumor microenvironment and the microbiota represent additional layers of redox regulation that remain underexplored.

Despite the promise of redox-targeted therapies, several challenges remain. First, validated biomarkers for predicting redox vulnerability are lacking, making patient stratification difficult. Second, ROS exhibit substantial spatial and temporal heterogeneity within tumors, and their effects depend on concentration, localization, and duration, thereby complicating therapeutic dosing. Third, tumors can activate compensatory antioxidant pathways (e.g., Nrf2, GSH, and Trx systems) when a single redox node is inhibited, limiting the efficacy of single-agent therapies. The effectiveness of redox-targeted therapy therefore depends strongly on tumor context. Such strategies may be particularly effective in tumors with high basal ROS levels, KEAP1/NFE2L2 alterations, GPX4 dependency, high SLC7A11 expression, or a hypoxic TME. Conversely, they may be less effective in tumors with low ROS levels, highly redundant antioxidant networks, marked intratumoral heterogeneity, or high target expression in normal tissues. Patient stratification and rational combination strategies will therefore be essential for successful clinical translation. Fourth, many pro-oxidant compounds have narrow therapeutic windows and may damage normal tissues. Fifth, delivery barriers, poor bioavailability, and off-target effects remain important obstacles to clinical translation. Finally, preclinical models often fail to fully recapitulate the complexity of the TME, and clinical trial design for combination regimens remains challenging. Addressing these issues will require improved biomarkers, rational combination strategies, and TME-informed patient selection.

In essence, the field is moving from viewing oxidative stress as a byproduct of tumor metabolism toward recognizing redox homeostasis as an organizing principle of cancer biology. Effective redox-based therapy will not simply involve increasing or decreasing ROS. Rather, it will depend on identifying context-specific vulnerabilities and intervening with sufficient precision to disrupt tumor survival networks while minimizing damage to normal tissues. Bridging the gap between mechanistic insight and actionable clinical strategies remains a major challenge for the field.

## Figures and Tables

**Table 1:** Natural ROS-modulating compounds.

Compound	Class	Molecular Structures	Clinical Applications	Ref.
Curcumin	Polyphenol	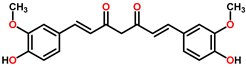	Colorectal, breast	[162]
Resveratrol	Stilbene	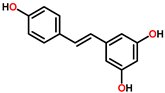	Colon, breast	[163]
Quercetin	Flavonoid	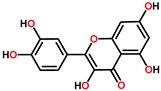	Lung, liver	[160]
Sulforaphane	Isothiocyanate	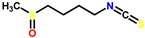	Breast, prostate	[161]
DHA	Sesquiterpene lactone derivative	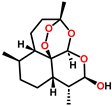	GBM, leukemia, lung, breast, liver	[164]
Thymoquinone	Monoterpene quinone	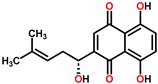	Multiple	[165]
Thymol	Monoterpene phenol	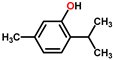	Breast, lung, colon	[166]
Triptolide	Diterpenoid epoxide	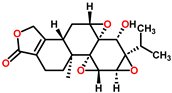	Pancreatic, leukemia, breast, lung	[167]
Betulinic acid	Polyphenol	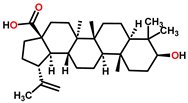	Liver, breast	[168]
Ursolic acid	Stilbene	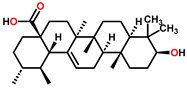	Breast, liver, leukemia	[169]
Shikonin	Flavonoid	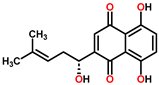	Prostate, breast, leukemia	[170]
Juglone	Isothiocyanate	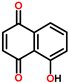	Leukemia, breast, lung	[171]
Rsl3	Iron-mediated death inducers	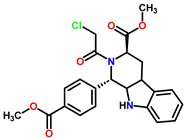	Colorectal Cancer, Breast Cancer, Bladder cancer	[172]

**Table 3:** Microbiome-related metabolites and redox strategies.

Compound	Class	Molecular Structures	Clinical Applications	Ref.
Riboflavin (Vitamin B2)	Microbiome-derived vitamin/redox cofactor	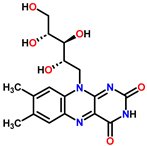	Multiple	[189]
Vitamin D	Secosteroid hormone	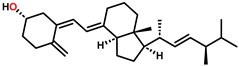	Colorectal, breast	[190]
Butyrate	SCFA metabolite	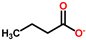	Colorectal	[193]
Deoxycholic acid (DCA)	Secondary bile acid		Multiple	[192]

**Table 4:** Cancer therapies that induce oxidative stress and their mechanisms.

Therapy Type	Examples	Mechanism of ROS Induction	Redox-Related Outcome	Ref.
Chemotherapy	Doxorubicin, cisplatin, paclitaxel	Mitochondrial ETC leakage, NADPH oxidase activation, GSH depletion	Apoptosis, ferroptosis, drug resistance	[197]
Radiotherapy	X-ray, γ-ray	Water radiolysis, ·OH generation, DNA damage	Apoptosis, senescence, immunogenic cell death	[185]
Immunotherapy	Anti-PD-1/PD-L1, CAR-T	T-cell activation, NOX2-derived ROS, cytokine release	T-cell exhaustion, immune evasion	[198]
Targeted therapy	Sorafenib, imatinib, EGFR inhibitors	Mitochondrial dysfunction, Nrf2/Keap1 modulation	Adaptive resistance, ferroptosis sensitization	[73]
Photodynamic therapy	Photofrin, 5-ALA	Photosensitizer-mediated ROS burst	Apoptosis, necrosis, ICD	[21]

## Data Availability

No new data were created or analyzed in this study. Data sharing is not applicable to this article.

## Abbreviations

The following abbreviations are used in this manuscript:

ROS	reactive oxygen species
TME	tumor microenvironment
NOX	NADPH oxidase
DUOX	dual oxidase
O_2_·^−^	superoxide anion
H_2_O_2_	hydrogen peroxide
ONOO^−^	peroxynitrite
·OH	hydroxyl radical
SOD	superoxide dismutase
SOD1	Cu/Zn-superoxide dismutase
SOD2	Mn-superoxide dismutase
SOD3	extracellular superoxide dismutase
PRDX	peroxiredoxin
GPX	glutathione peroxidase
GPX4	glutathione peroxidase 4
GST	glutathione S-transferase
GSH	reduced glutathione
GSSG	oxidized glutathione
Trx	thioredoxin
TrxR	thioredoxin reductase
NADPH	nicotinamide adenine dinucleotide phosphate (reduced form)
PPP	pentose phosphate pathway
G6PD	glucose-6-phosphate dehydrogenase
xCT	cystine/glutamate antiporter
SLC7A11	solute carrier family 7 member 11
Nrf2	nuclear factor erythroid 2–related factor 2
Keap1	Kelch-like ECH-associated protein 1
ARE	antioxidant response element
HO-1	heme oxygenase-1
NQO1	NAD(P)H quinone dehydrogenase 1
NF-κB	nuclear factor kappa B
IκB	inhibitor of κB
IKK	IκB kinase
FHC	ferritin heavy chain
MAPK	mitogen-activated protein kinase
ERK	extracellular signal-regulated kinase
JNK	c-Jun N-terminal kinase
MKP	MAPK phosphatase
PI3K	phosphoinositide 3-kinase
AKT	protein kinase B
mTOR	mechanistic target of rapamycin
PTEN	phosphatase and tensin homolog
ER	endoplasmic reticulum
ERO1	endoplasmic reticulum oxidoreductin 1
PDI	protein disulfide isomerase
UPR	unfolded protein response
XDH	xanthine dehydrogenase
XO	xanthine oxidase
EMT	epithelial–mesenchymal transition
ECM	extracellular matrix
CAFs	cancer-associated fibroblasts
TAM	tumor-associated macrophage
Treg	regulatory T cell
PCD	Programmed cell death
DDR	DNA damage response
ATM	ataxia telangiectasia mutated
ATR	ataxia telangiectasia and Rad3-related
DDS	drug delivery system
HIF-1α	hypoxia-inducible factor-1 alpha
IL-6	interleukin 6
TNF-α	tumor necrosis factor alpha
VEGF	vascular endothelial growth factor
TMZ	temozolomide
GBM	glioblastoma multiforme
